# Supplementary material for: Photoregulated fluxional fluorophores for live-cell super-resolution microscopy with no apparent photobleaching
Source: Nat Commun. 2019 Mar 15;10:1232. doi: 10.1038/s41467-019-09217-7 (PMC6420572; doi:10.1038/s41467-019-09217-7)
Supplement: Supplementary file 1 — Supplementary Information [file 41467_2019_9217_MOESM1_ESM.pdf]

Supplementary Information for

Photoregulated Fluxional Fluorophores for Super-Resolution Microscopy with no Apparent Photobleaching

Elias A. Halabi, Dorothea Pinotsi, and Pablo Rivera-Fuentes*

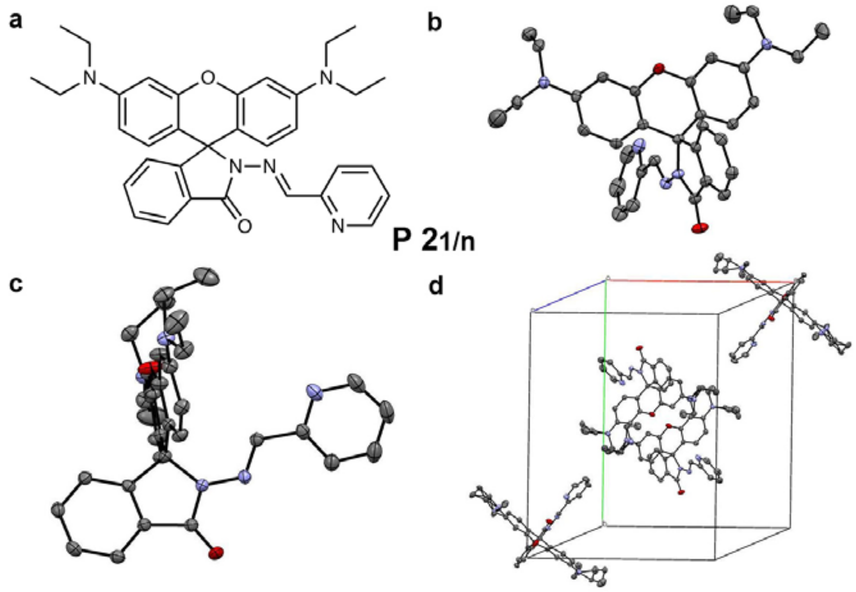

Supplementary Figure 1 | Solid-state structures obtained by single-crystal X-ray crystallography. **a**, Molecular structure of compound PFF-**1**. **b**, Front view. **c**, Side view. **d**, Unit cell. H-Atoms as well as special positions C31A, C33, C28A, and C1A were omitted for clarity. Atomic displacement parameters at 100 K are drawn at 50% probability level. Crystallographic data for PFF-**1** can be found at the Cambridge Crystallographic Data Centre under CCDC no. 1844696.

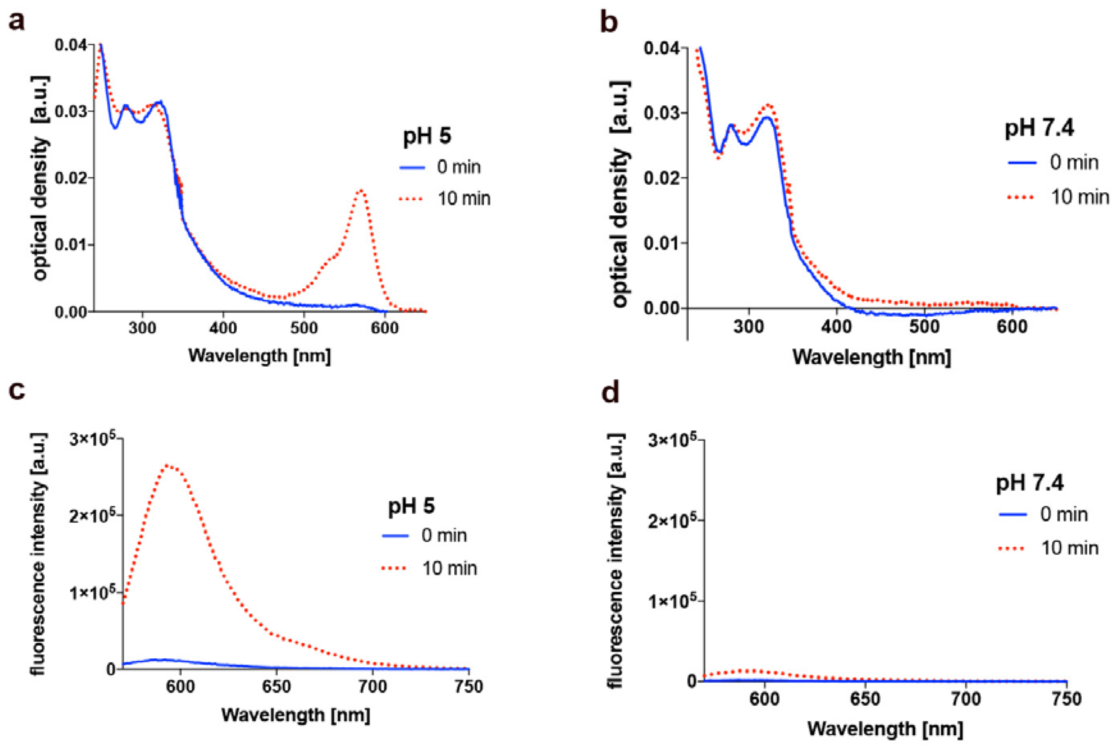

Supplementary Figure 2 | Photoactivation of compound PFF-1 in cuvettes. **a**,**b**, UV-Vis spectra of compound PFF-**1** (1 μM) before irradiation (0 min) and after irradiation (10 min) at pH = 5 and pH = 7.4 respectively. **c**,**d**, Fluorescence spectra of compound PFF-**1** (1 μM) before irradiation (0 min) and after irradiation (10 min) at pH = 5 and pH = 7.4 respectively. All measurements were carried out in phosphate-citrate buffer at the indicated pH and irradiation was performed with an LED trans-illuminator (410 nm, 2 mW cm^{-2}). The quantum yield of emission of compound PFF-**1** in its emissive form is 17±3% at pH = 5.

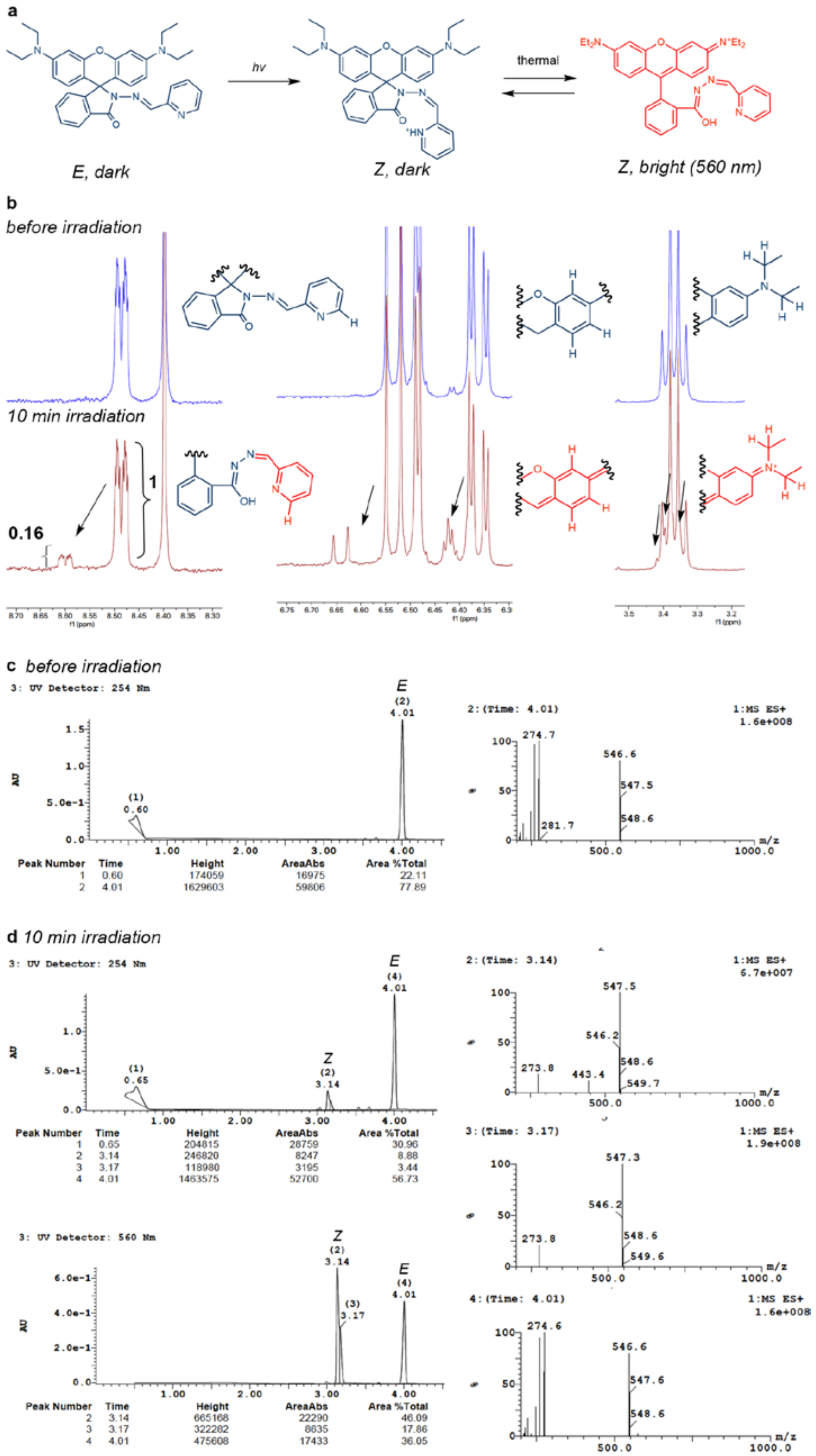

Supplementary Figure 3 | Photoirradiation experiments of PFF-1 in CH_3CN followed by ^{1}H NMR and LC-MS. a. Scheme of the photoisomerization of PFF-**1**

from the *Z to E* isomer. **b**.^{1}H NMR spectrum in CD_3CN before (blue) and after (red, 10 min, 375 nm lamp) irradiation. In the aromatic region, (8.65–8.45 ppm) the shift of the highlighted pyridyl hydrogen is promoted by the interaction with the carbonyl oxygen upon isomerization. Additional aromatic protons (6.70–6.3 ppm) exhibit a shift corresponding to the change in conjugation system within the xanthene core. Finally, the aliphatic region (3.5–3.3 ppm) displays a slight shift in the alkyl protons upon formation of the partially charged nitrogen species. The integration of the respective peaks correlates to approximately 16% conversion (*E* → *Z*). LC-MS trace (CH_3CN:H_2O with 0.1% formic acid) of a solution of PFF-**1** in CH_3CN before **c** and after **d** irradiation (10 min, 375 nm lamp). The *Z* isomer exists predominantly (>99%) and can be monitored at 254 nm and 560 nm. At 560 nm, the *Z* isomer has a much higher absorbance than isomer *E*. After irradiation, an approximate conversion (17.8% *E* → *Z*) was estimated using the area under the curve monitored at 254 nm. All isomers could be identified by mass spectrometry (546.6 m/z) and retention time.

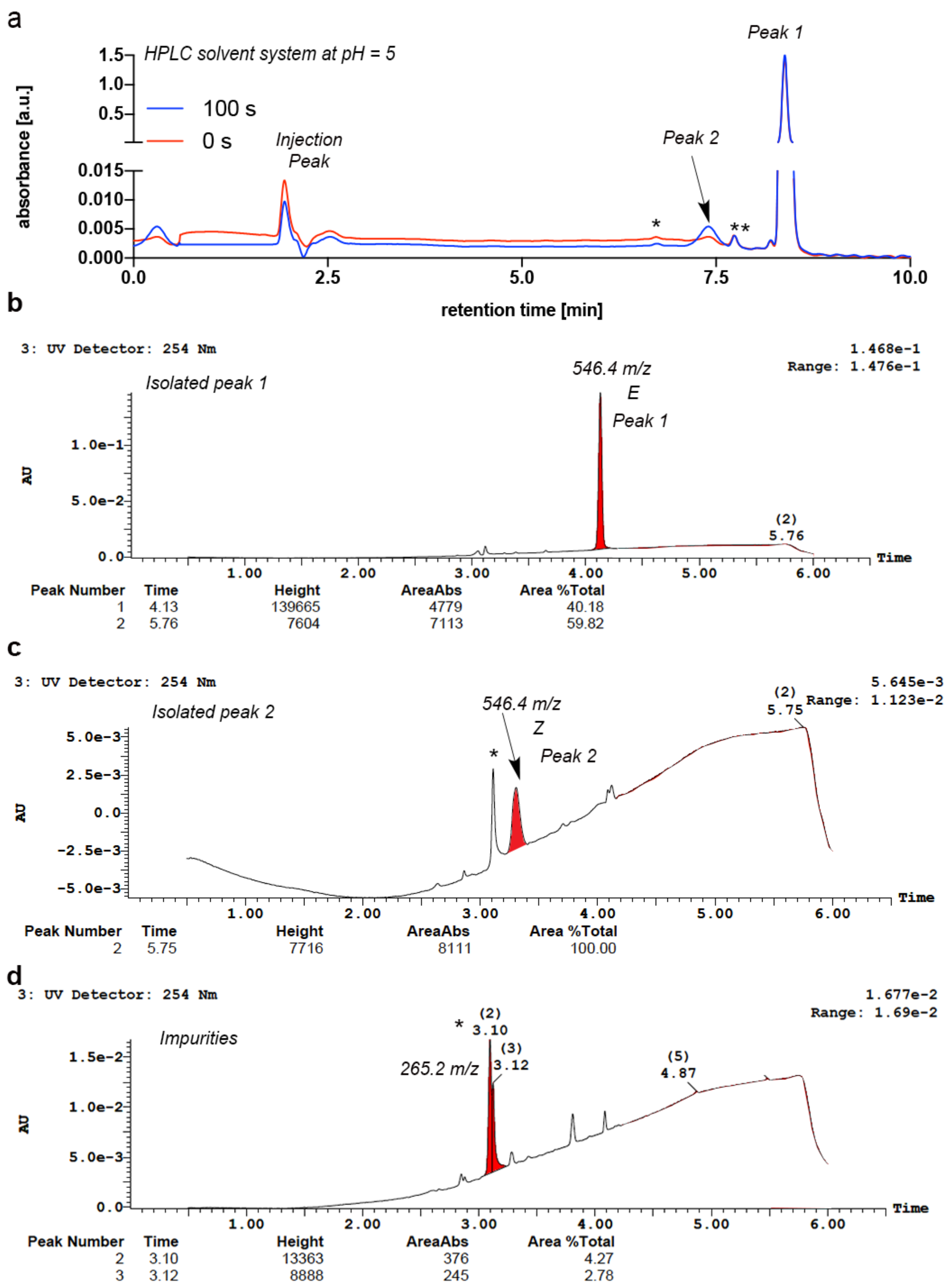

Supplementary Figure 4 | HPLC analysis of the *E* and *Z* isomer at pH = 5. a, HPL chromatogram (citric acid + Na_2HPO_4 buffer:CH_3CN, pH = 5, 50:50 → 100%) of a PFF-**1** solution (50 μM, citric acid + Na_2HPO_4 buffer:CH_3CN, pH = 5) before (*t* = 0

s) and after irradiation (*t* = 100 s) using a 405 nm LED light source. The chromatogram monitored at 254 nm shows the presence of the *E* isomer (peak 1), the *Z* isomer (peak 2) identified from the isolated fraction by LC-MS (**b** and **c** respectively). **d**. Small amount of unidentified impurities (*,**) were observed prior to irradiation and were not produced during the photoreaction.

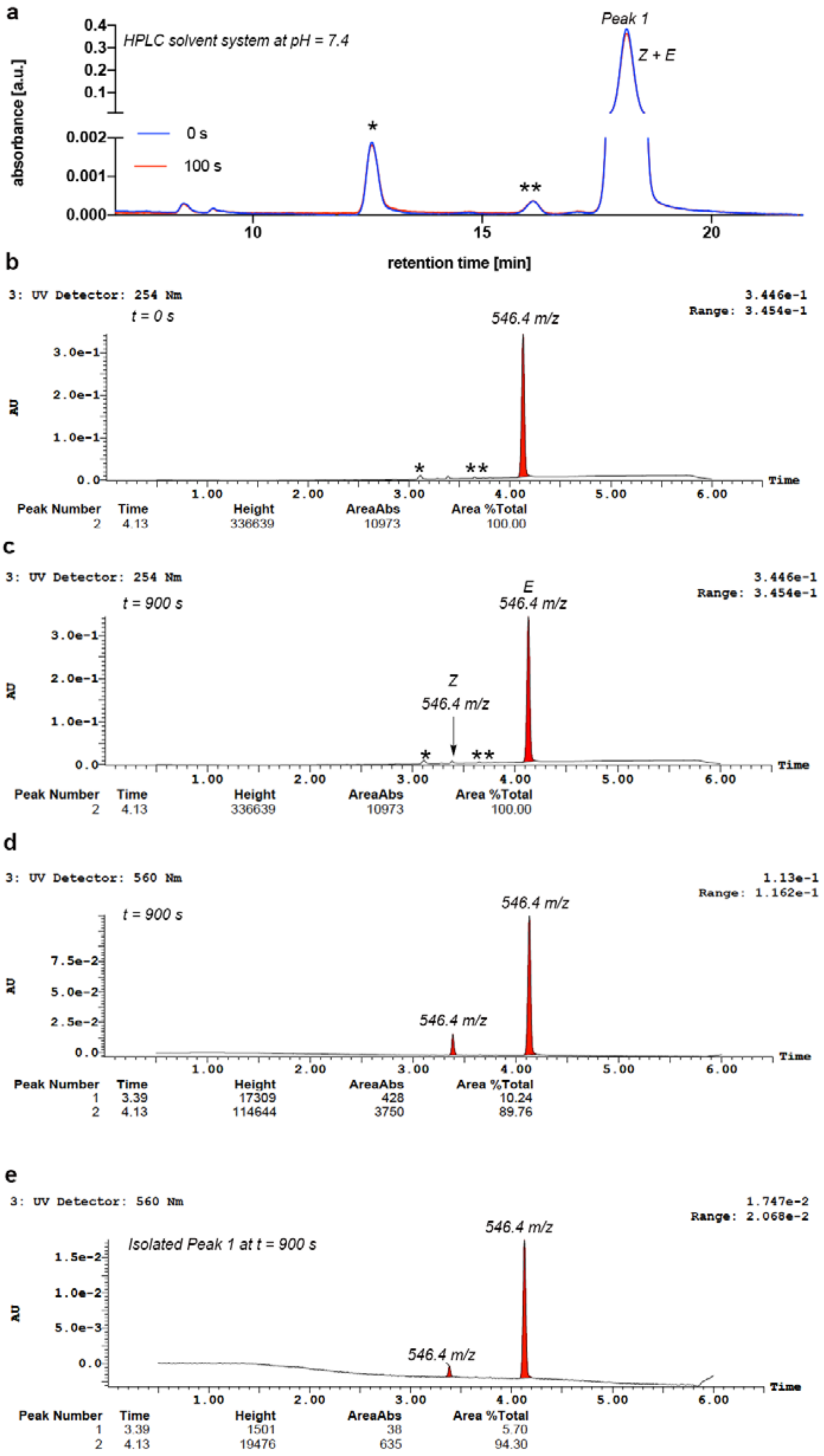

Supplementary Figure 5 | Separation of the *E* and *Z* isomer by HPLC at pH = 7.4. a. HPL chromatogram (citric acid + Na_2HPO_4 buffer:CH_3CN, pH = 7.4, 50:50 → 60%) of a PFF-**1** solution (20 μM, citric acid + Na_2HPO_4 buffer:CH_3CN, pH = 7.4)

before and after irradiation (*t* = 100 s) using a 405 nm LED light source. The chromatogram monitored at 254 nm reveals the presence of only one peak (mixed isomers) and two unidentified impurities (*,**) detected by LC-MS (**b**). LC-MS traces of the same solution subjected to 900 s of irradiation monitored at 240 nm (**c**) and 560 nm (**d**). The isolated fraction from the HPLC run, containing peak 1 (*t* = 900 s, **e**) suggesting that both isomers were not separable at pH = 7.4 (LC-MS analysis was carried out in the presence of 0.1% formic acid). These data suggest that protonation induced fluxionality of the *Z* isomer promotes a significant shift in retention time.

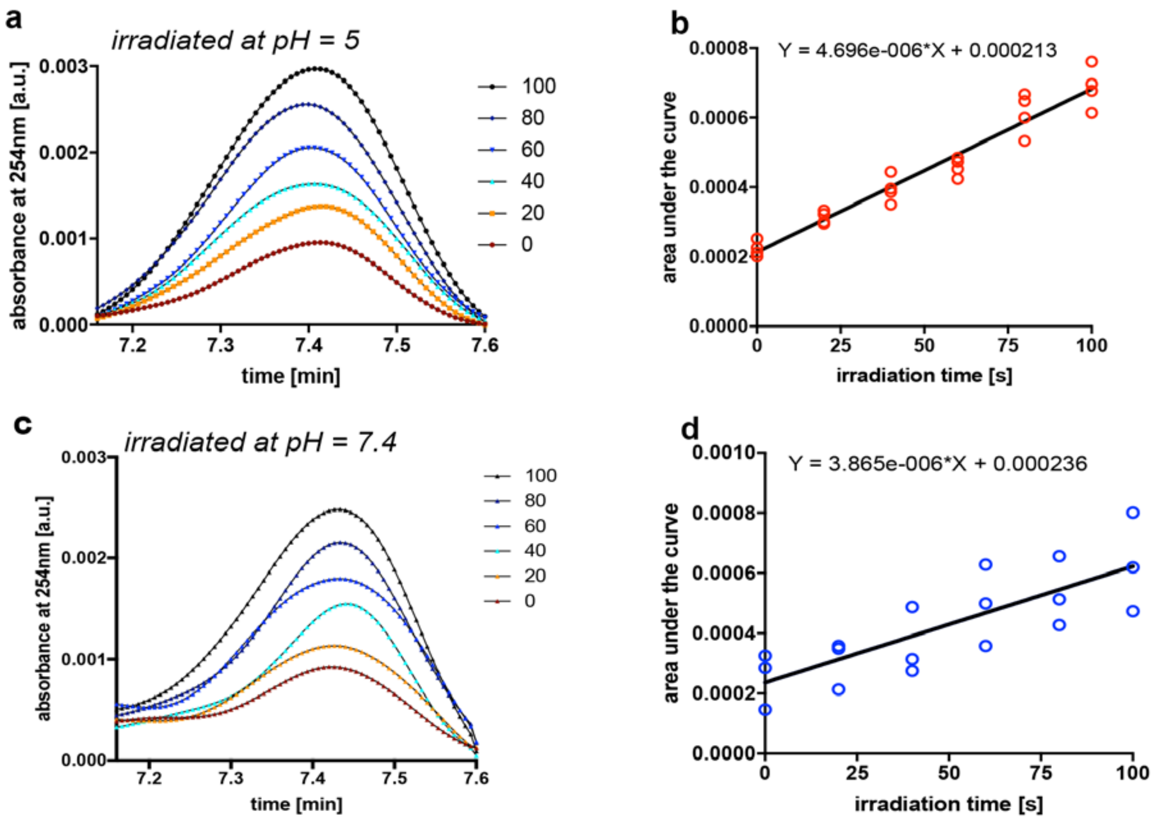

Supplementary Figure 6 | Kinetics of photoisomerization of PFF-1. HPLC analysis (citric acid + Na_2HPO_4 buffer:CH_3CN, pH = 5, 50:50 → 100%) of a PFF-**1** solution (50 μM, citric acid + Na_2HPO_4 buffer:CH_3CN, pH = 5) irradiated in time intervals (t = 0, 20, 40, 60, 80 and 100 s) using a 405 nm LED light source and monitored at 254 nm. **a** and **b**, HPL chromatogram and linear regression plots of the peak corresponding to the Z isomer irradiated at pH = 5. **c** and **d,** HPL chromatogram and linear regression plots of the peak corresponding to the Z isomer irradiated at pH = 7.4. Open circles represent experimental points (triplicate) and the slope was obtained from the calculated linear regressions.

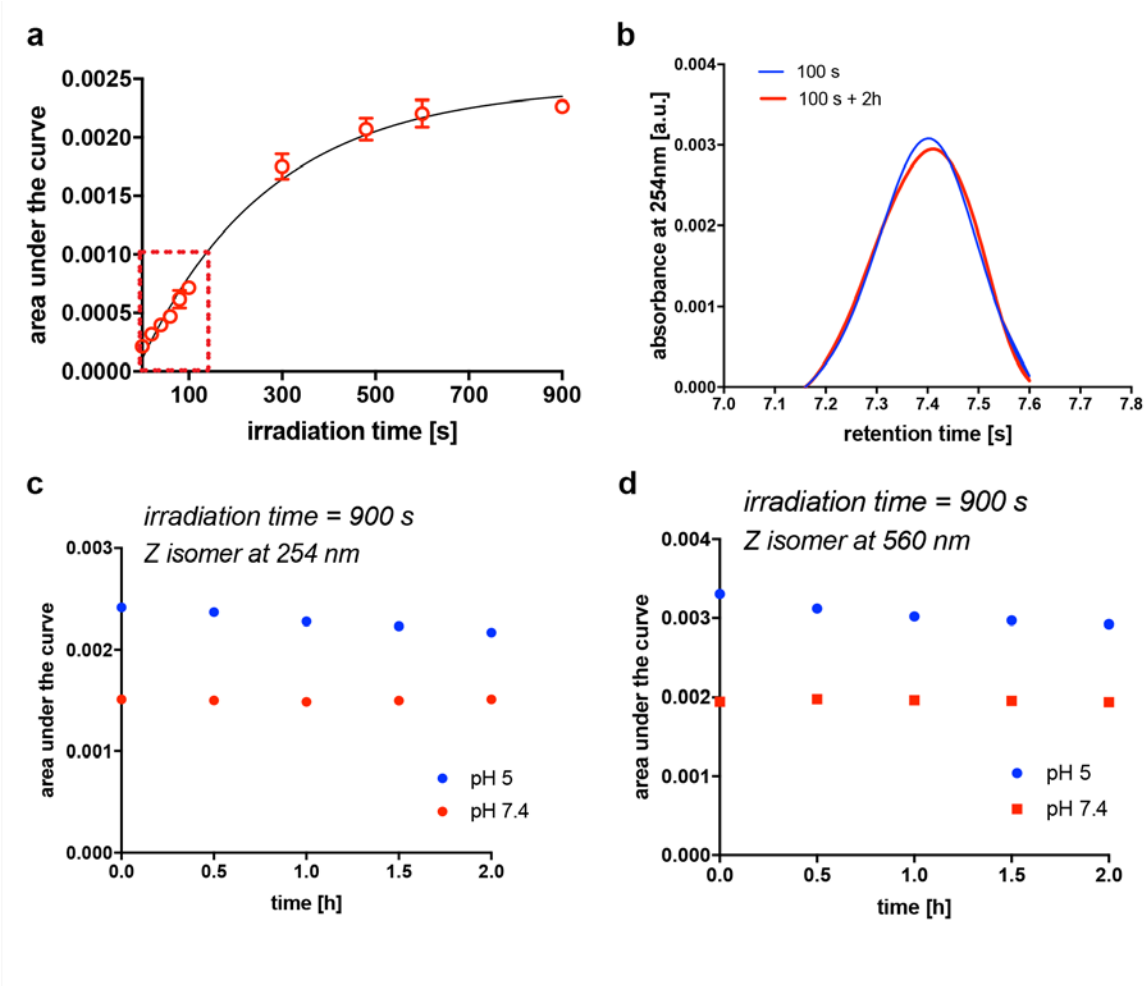

Supplementary Figure 7 | Photostationary state and thermal stability of PFF-1. **a,** Plot of the area under the curve of the peak corresponding to the *Z* isomer in the HPL chromatogram at different irradiation times of a solution of PFF-**1** in citric acid + Na_2HPO_4 buffer:CH_3CN (pH = 5) after irradiation with 405 LED (t = 0, 20, 40, 60, 80, 100, 300, 450, 600 and 900 s). The dotted red square represents the linear regime (< 150 s irradiation time) and a photostationary state was reach at ~900 s of irradiation (405 nm, LED) monitored at 254 nm. Open circles represent experimental points (mean and standard deviation of triplicates) and the line is the fit to an exponential one phase decay. **b**. HPL chromatogram of the peaks corresponding to the *Z* isomer after 100 s of irradiation and of the same solution after 2 h incubation time in the dark. **c** and **d**, Stability measurements for the *Z* isomer of PFF-**1** after

reaching the photostationary state (900 s) at pH = 5 and pH = 7.4 monitored in both wavelengths (254 and 560 nm). The points are the area under the curve of the corresponding peak in the HPL chromatogram measured at the corresponding wavelength.

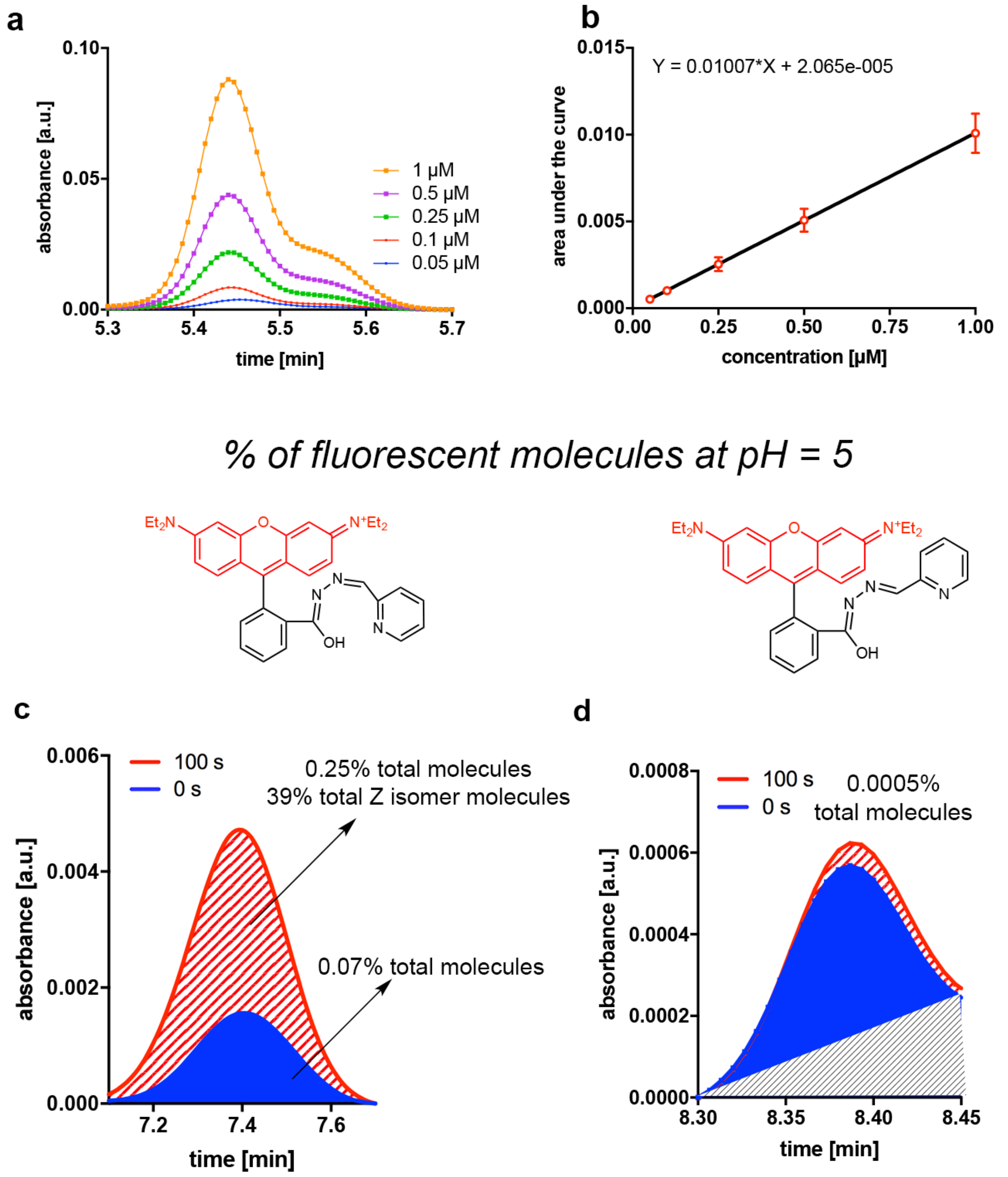

Supplementary Figure 8 | Quantification of total ON fraction of the *E* and *Z* isomers. a. HPL chromatogram of the peak corresponding to rhodamine B (pH = 1) at 560 nm. A total of five concentrations were measured (0.05, 0.1, 0.25, 0.5 and 1 μM) employing the same HPLC method (monitored at 560 nm). **b**. Calibration curve

built using the area under the curve for the solutions measured in panel **a**. Open circles with error bars represent experimental points (mean and standard deviation of triplicates). The obtained slope was used for estimating the concentration (μM) of fluorescent PFF-**1** molecules assuming that they have the same absorbance as rhodamine B at pH = 1. **c** and **d**, HPL chromatogram of the fluorescent molecules of *Z* and *E* isomer before (*t* = 0 s) and after irradiation (*t* = 100 s) irradiated at pH = 5, respectively

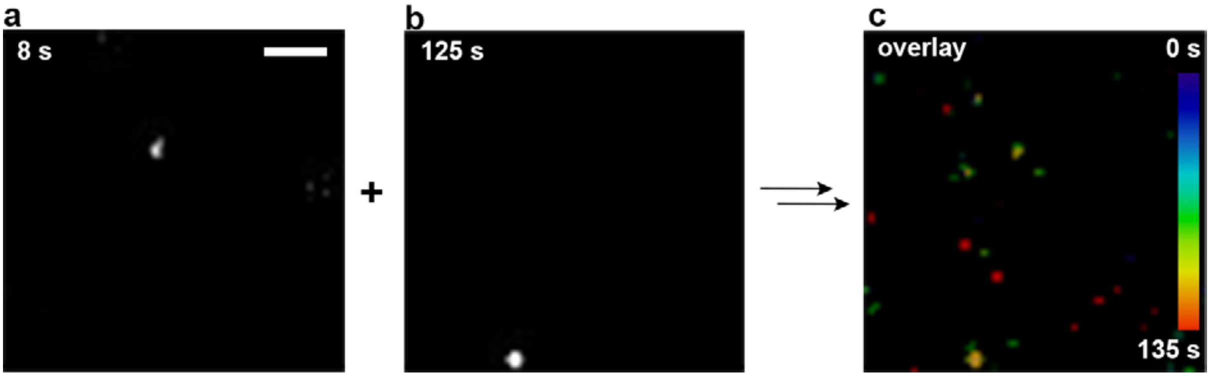

Supplementary Figure 9 | Single-molecule imaging of compound 2. a-b. Single molecules emitting at 8 and 125 s respectively. **c**. Overlay of the total 135 s (6500 frames) collected for the acquisition and color coded according to time. Most of the colors in the color-coded image display a single color because most molecules emit photos at a single time point. Activation pulses (10 ms) were performed every 200 frames with a 405 nm laser light (2.6 W cm^{-2}). Emission read-out was obtained from 561 nm laser (0.25 kW cm^{-2}) with a 20 ms exposure time and 48 frame per second camera rate.

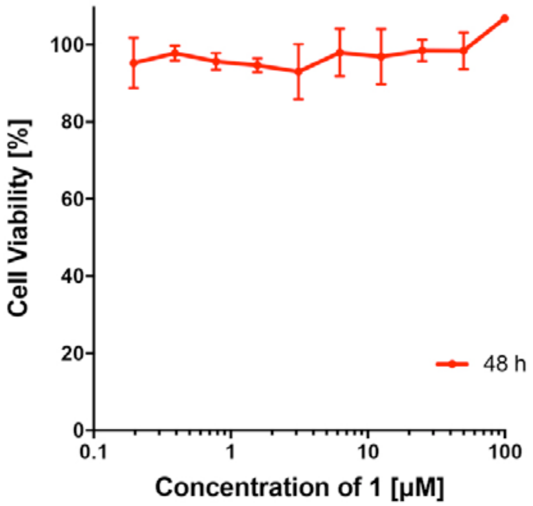

Supplementary Figure 10 | Plot of cell viability (MTT) against concentration of compound PFF-1 after 48 h of incubation (for details, see the methods section). Error bars represent experimental points (mean and standard deviation of triplicates)

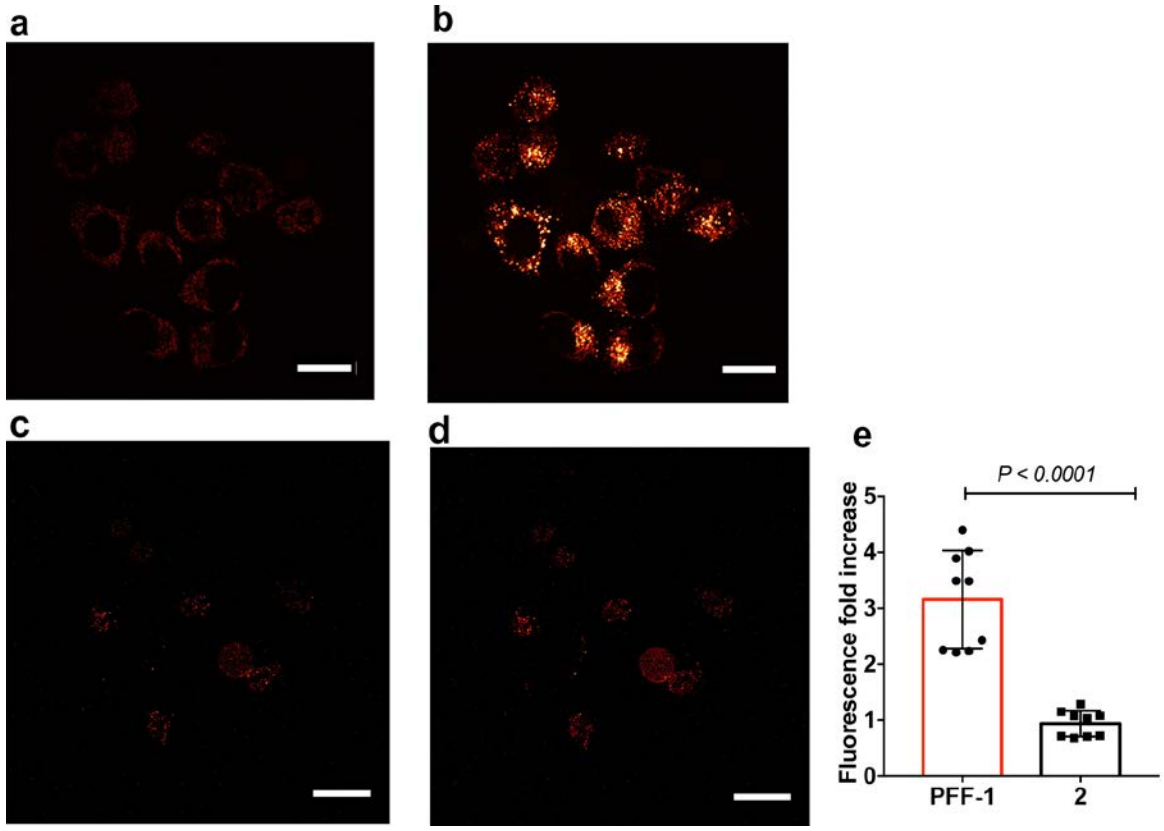

Supplementary Figure 11 | Photoactivation experiments in live HeLa cells. **a**,**b**, Cells were incubated with compound PFF-**1** (10 μM) and imaged with 561 nm laser excitation (140 mW cm^{-2}, 0.5 s) before and after photoactivation (405 nm, 600 mW cm^{-2}, 1 s), respectively. **c**,**d,** Cells were incubated with compound **2** (10 μM) and imaged with 561 nm laser excitation (140 mW cm^{-2}, 0.5 s) before and after photoactivation (405 nm, 600 mW cm^{-2}, 1 s), respectively. **e**. Photoactivation efficiency of PFF-**1** and **2** in HeLa cells using the same imaging conditions as in panel **a**-**d**. Scale bars for **a**,**b** = 10 μm.

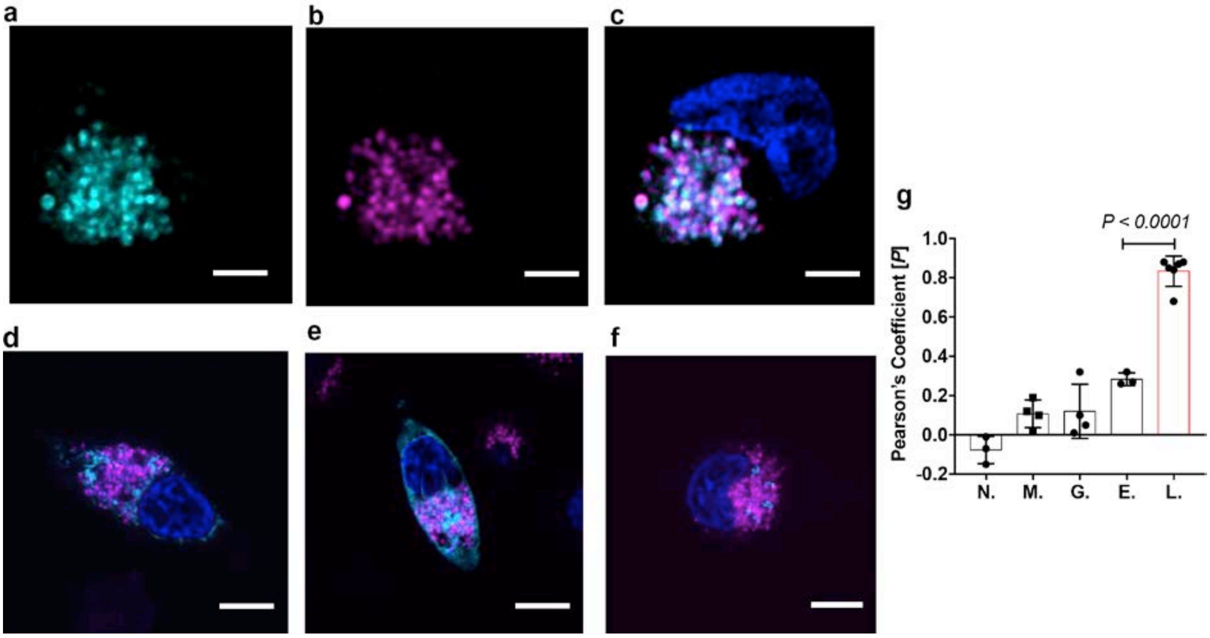

Supplementary Figure 12 | Co-localization in live HeLa cells. **a**,**b**, Fluorescent signals obtained from a HeLa cell transfected with pLAMP1-mTurquoise2 plasmid (green) and incubated with compound PFF-**1** (10 μM, red), respectively. **c**, Overlay of mTurquoise2 and probe PFF-**1** emission from panels **a** and **b**. **d**-**f**, Co-localization experiments of compound PFF-**1** (10 μM) with mTurquoise2-Mitochondria, mTurquoise2-ER, and mTurquoise2-Golgi respectively. Nuclei were stained with Hoechst 33342 (10 μM). Activation was carried out with 405 nm laser (60 mW cm^{-2}, 500 ms), read out with a 445 nm laser for mTurquoise2 (100 mW cm^{-2}, 0.5 s) and a 561 nm laser for compound PFF-**1** (100 mW cm^{-2}, 0.5 s). **g**, Pearson's correlation coefficient (*P)* values for the co-localization of compound PFF-**1** with nucleus (N), mitochondria (M), Golgi apparatus (G), endoplasmic reticulum (E), and lysosomes (L). Mean and standard deviation of at least N = 3 measurements from biological triplicate are depicted. Statistical significance was assessed by unpaired *t*-test and two-tailed *P* value is provided. Scale bars **a**-**f** = 5 μm.

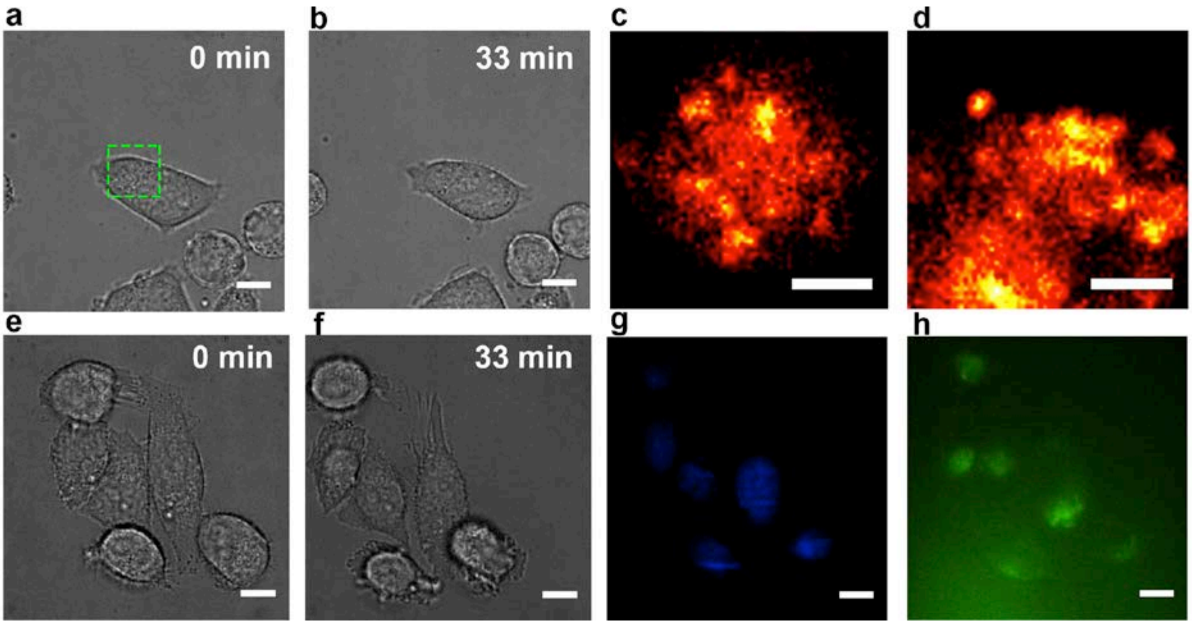

Supplementary Figure 13 | Assessment of phototoxicity after long-term, time-lapse experiments. **a**,**b**, Bright-field images of a HeLa cell before (0 min) and after (33 min) prolonged 2D SMLM imaging, respectively. **c**,**d**, Zoom-in region (green square in panel **a** of the HeLa cell imaged in 561 nm channel (1.2 kW cm^{-2}, 20 ms) before and after long-term imaging respectively. **e**,**f**, Brightfield images of a HeLa cell before (0 min) and after (33 min) prolonged 2D SMLM imaging respectively. **g**,**h**, Same cells as in panel **f** incubated with 4',6-diamidino-2-phenylindole (DAPI, 10 μM, 10 min, membrane permeabilization indicator) and imaged in 405 nm channel (2.6 W cm^{-2},10 ms) and CellTox™ Green Dye (10 μM, 10 min, capase-3 activation assay) imaged in 488 nm channel (0.18 kW cm^{-2}, 10 ms) respectively. Scale bars for **a**, **b**, **e**-**h** = 10 μm and **c**, **d** = 3 μm.

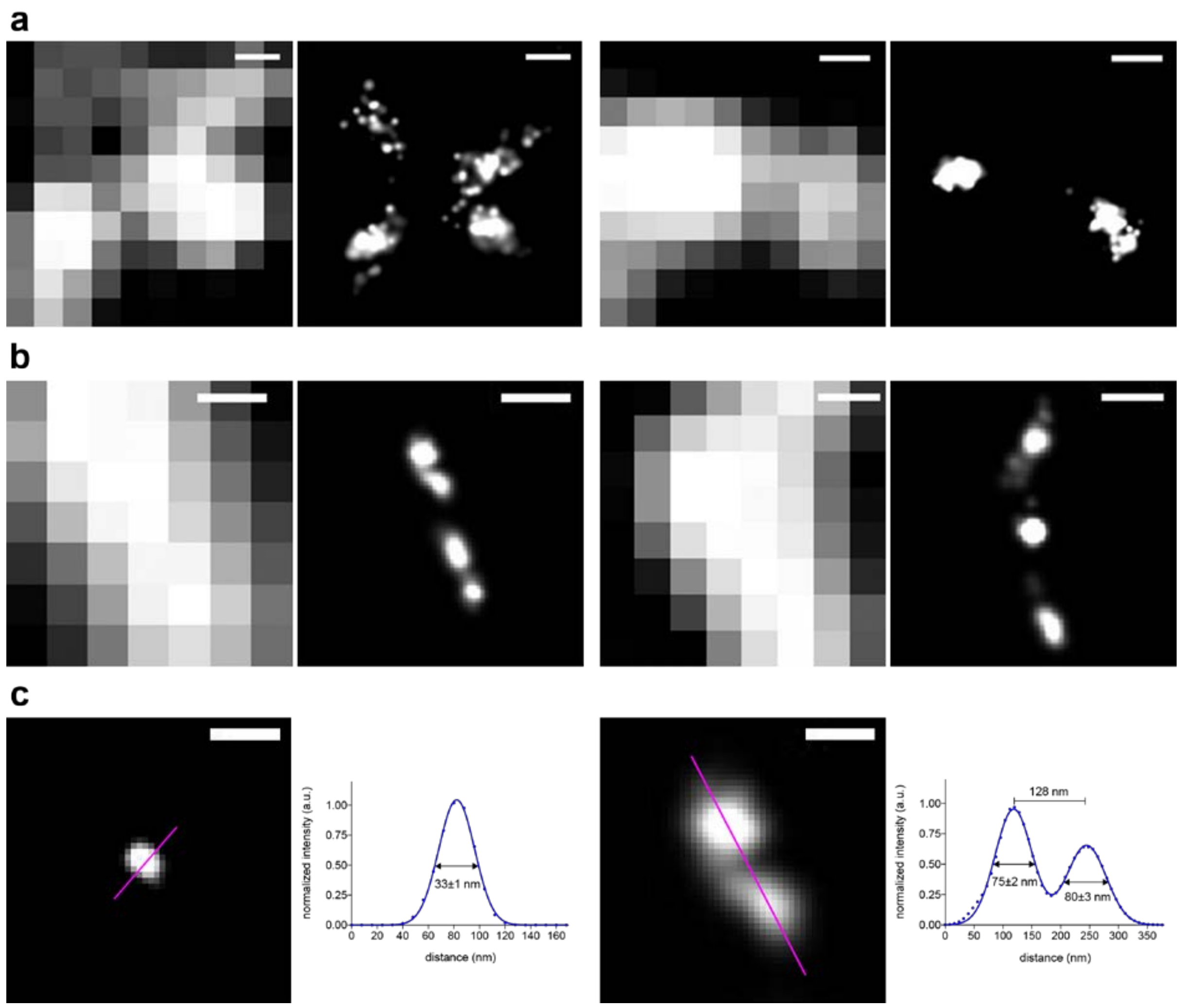

Supplementary Figure 14 | Super-resolved structures of lysosomes and synaptic vesicles. **a**, Comparison of diffraction-limited (HiLO) and super-resolved images of two groups of lysosomes in close proximity to each other. **b**, Comparison of diffraction-limited (HiLO) and super-resolved images of two groups of synaptic vesicles in close proximity to each other. **c**, Examples of super-resolved synaptic vesicles. The magenta lines indicate the path used for the intensity profiles in the plots. The pixel values along the lines were fit to a Gaussian distribution (R^2 > 0.99 in both cases). Full-widths at half-maximum (FWHM) were calculated from the standard deviation of the Gaussian fit. Scale bars for **a**, **b** = 250 nm and **c**, = 100 nm.

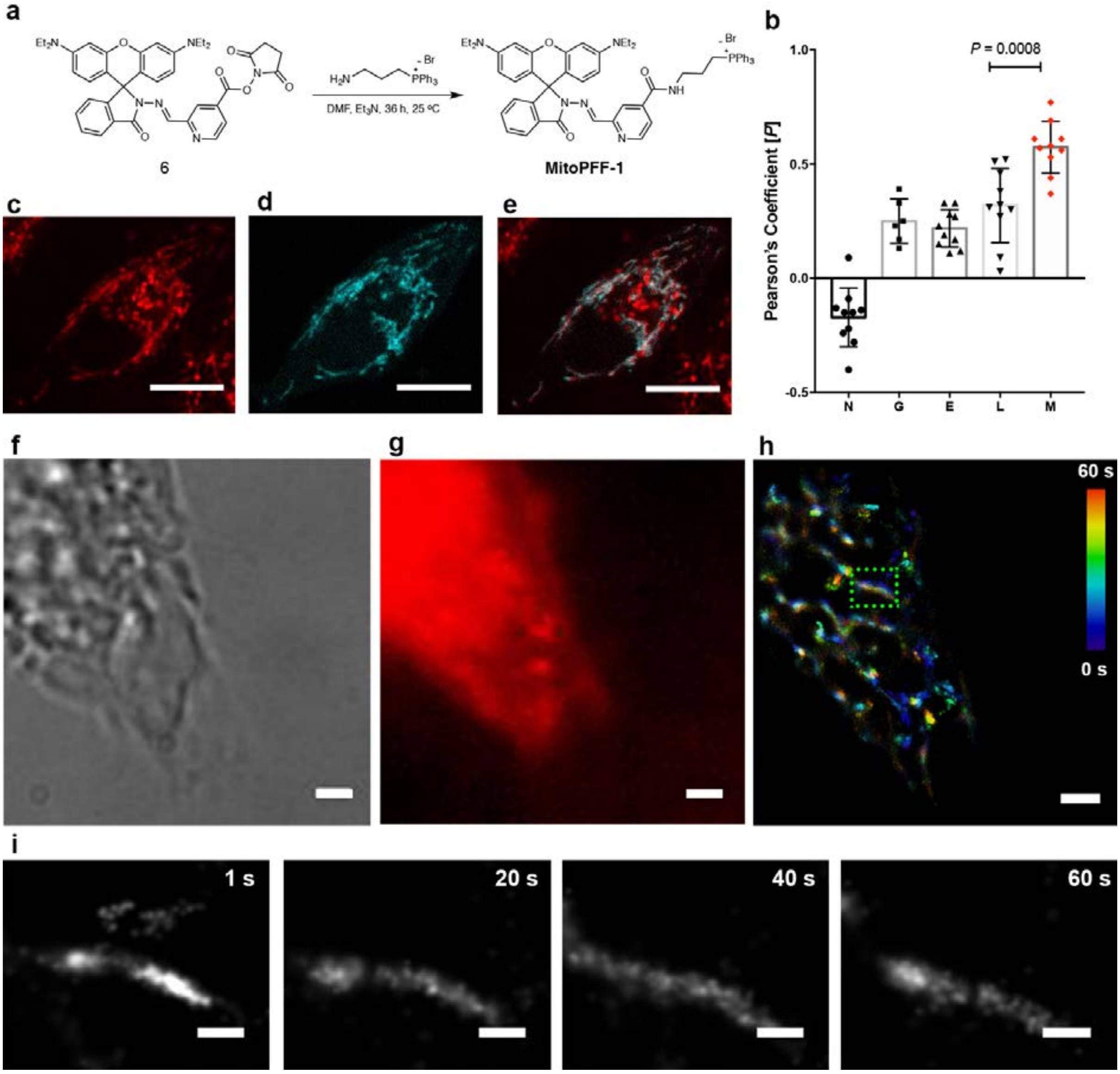

Supplementary Figure 15 | Colocalization and super-resolution imaging of MitoPFF-1 in HeLa cells. a, Synthesis scheme and structure of MitoPFF-**1**. **b**, Pearson's correlation coefficient (*P)* values for the co-localization of compound MitoPFF-**1** with nucleus (N), mitochondria (M), Golgi apparatus (G), endoplasmic reticulum (E), and lysosomes (L). Mean and standard deviation of at least N = 10 measurements from biological triplicate are depicted. Statistical significance was assessed by unpaired *t*-test and two-tailed P value is provided. **c**,**d**, Fluorescent signals obtained from a HeLa cell transfected with pmTurquoise2-Mito plasmid

(cyan) and incubated with compound MitoPFF-**1** (10 μM, red), respectively. **e**, Overlay of mTurquoise2 and probe MitoPFF-**1** emission from panels **c** and **d**. **f**, Brightfield image of a HeLa cell incubated with MitoPFF-**1** (10 μM). **g**, Diffraction limited image of the cell in panel **e** (overlay of 6000 frames). **h**, Super-resolution of the same frames in panel **g** (2 s time resolution, 30 frames) colored in time (1-60 s). The green dotted square indicates the area displayed in panel **i**. **i**, Time lapse of a mitochondria within 1 min of acquisition (2 s time resolution). Scale bars **c**-**d** = 5 μm, **f**-**h** = 2 μm and **i** = 500 nm.

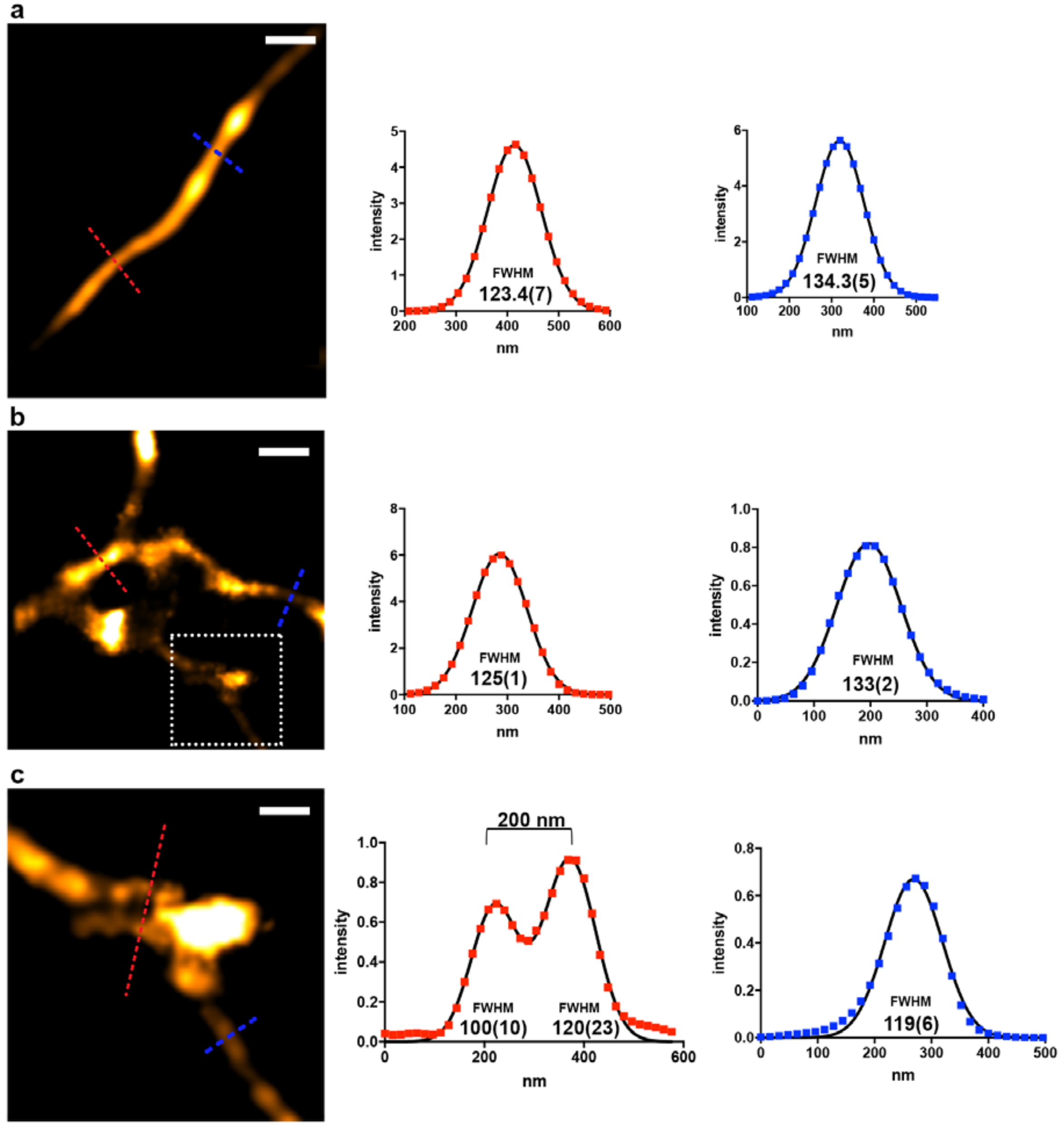

Supplementary Figure 16 | Super-resolution imaging of extracted microtubules using TaxolPFF-1. HeLa cells were cultured, permeabilized (PBS-0.1% Triton X-100, 15 s), fixed with methanol (–20 ^{o}C, 3 min), and incubated with a solution of TaxolPFF-**1** (10 μM in PBS, 1 h). Cells were washed thoroughly with phosphate buffer (pH = 5) and the extracted microtubular structures were imaged at pH = 5. **a.** Reconstructed image of a single microtubule using 12000 frames (120 s, camera rate 100 frames s^{-1}). Two regions of the microtubules were selected and the FWHM was calculated by fitting the obtained intensity curve to a Gaussian distribution and

multiplying the standard deviation (σ) by 2.35. **b**. Additional example of a microtubule network showing a region of interest (ROI). **c**. ROI of panel **b** showing two microtubules contained within the diffraction limit and resolved. The FWHM was determined by fitting the intensity points to a sum of Gaussian functions. Scale bars **a** and **b**; 500 nm, **c**; 200 nm.

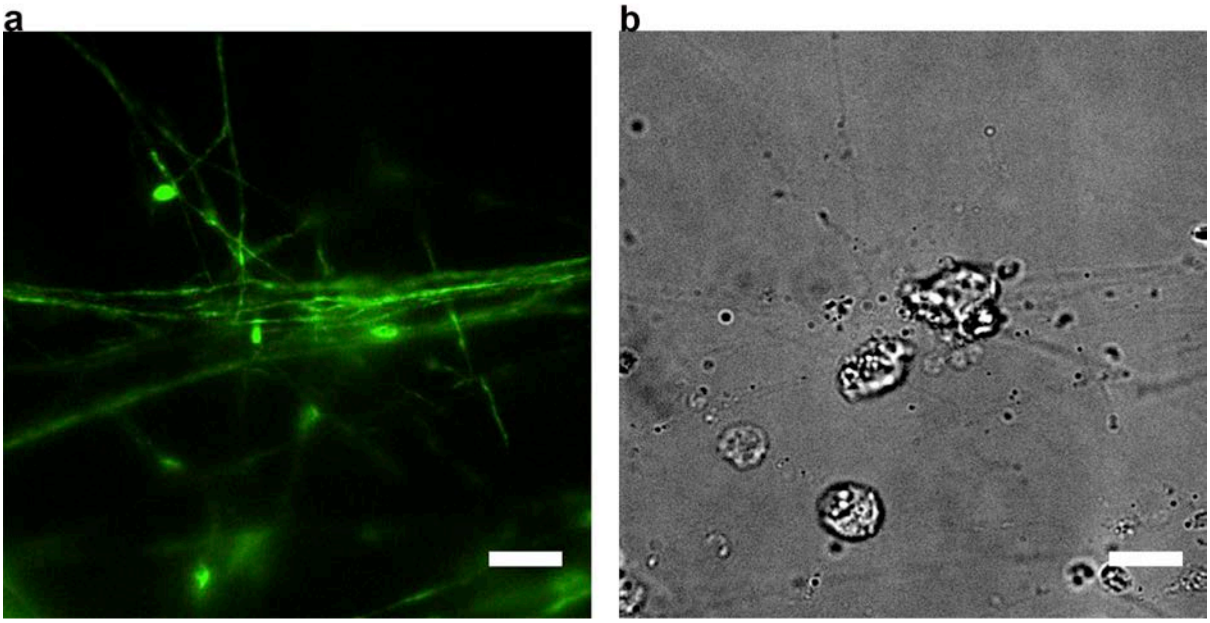

Supplementary Figure 17 | Marker of neuronal differentiation. a, Neurons treated with anti-SMI31 selectively staining the phosphorylated neurofilament H in neurites and imaged with 488 nm laser (0.18 kW cm^{-2}, 20 ms). **b**, Corresponding bright-field image of panel **a**. Scale bars = 10 μm.

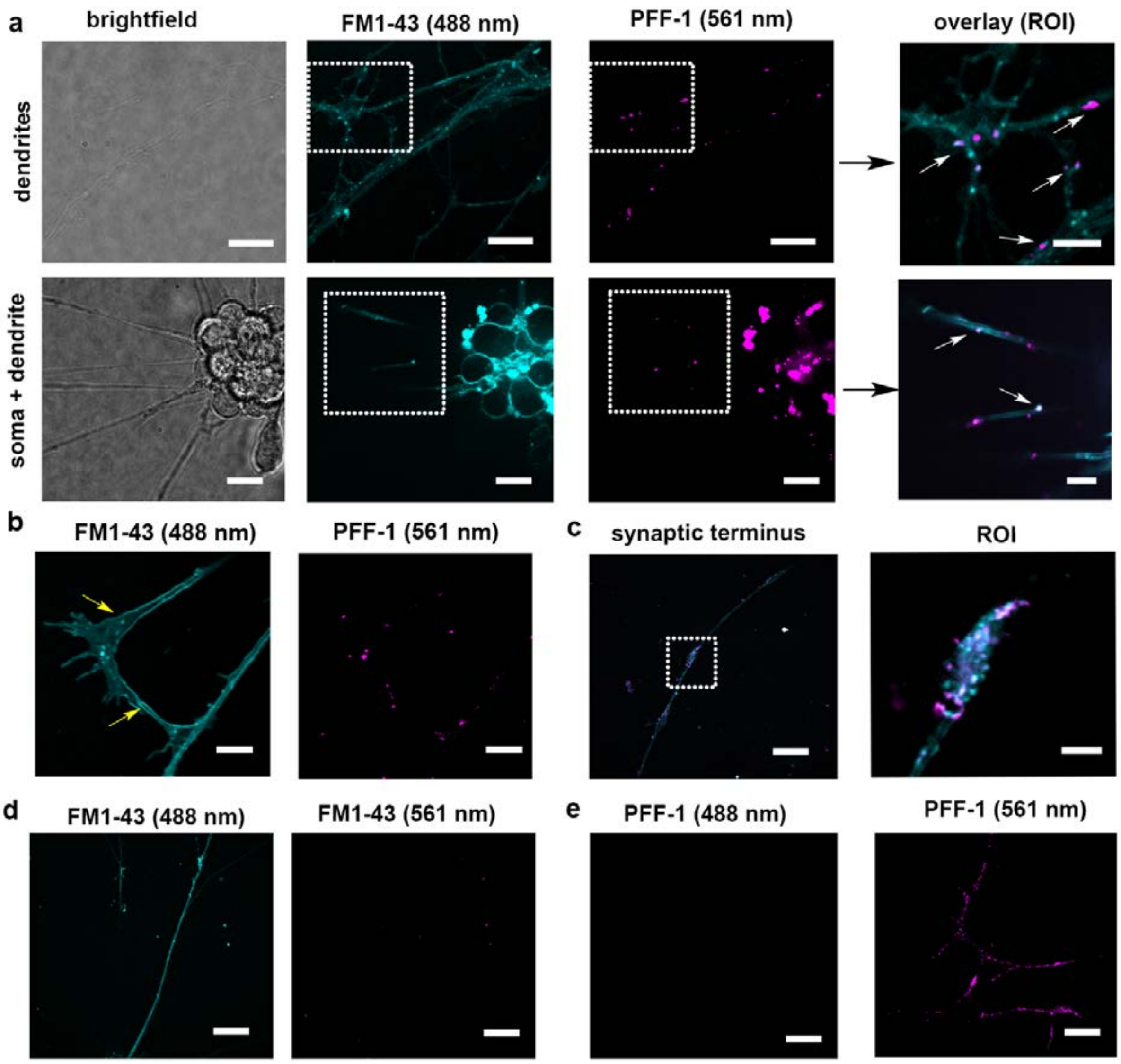

Supplementary Figure 18 | Colocalization of synaptic vesicles stained with FM1-43 and PFF-1. a, Imaging of dendrites (top) and somas (bottom) of fully mature mammalian neurons incubated with FM1-43 and PFF-**1** (10 μM). Images were acquired in the green (488 nm, 200 ms) and red (561 nm, 300 ms) channels after photoactivation. A ROI (white square) was selected and depicted as a merged image displaying the group of synaptic vesicles that appear in both channels (white arrows). **b**, Image of a living neuron under the same staining and imaging conditions as in panel **a**, depicting the stained plasma membrane (yellow arrows) using FM1-43, selective staining of intracellular acidic vesicles using PFF-**1**. **c**, Image of a living

neuron under the same staining and imaging conditions as in panel **a**, depicting a synaptic terminus with a high degree of colocalization in both channels. **d**, Additional case of specific co-localization of PFF-**1** to vesicles and staining of the plasma membrane by FM1-43. **e**, Demonstration that PFF-**1** does not produce a signal in the green imaging channel (488 nm). Scale bars **a**-**c** = 5 μm, ROI = 2.5 μm, **d**-**e** = 10 μm. *Note:* FM1-43 labels the cellular membrane and is taken up by vesicles during recycling, therefore not all synaptic vesicles necessary contain FM1-43, as observed in some of the images.

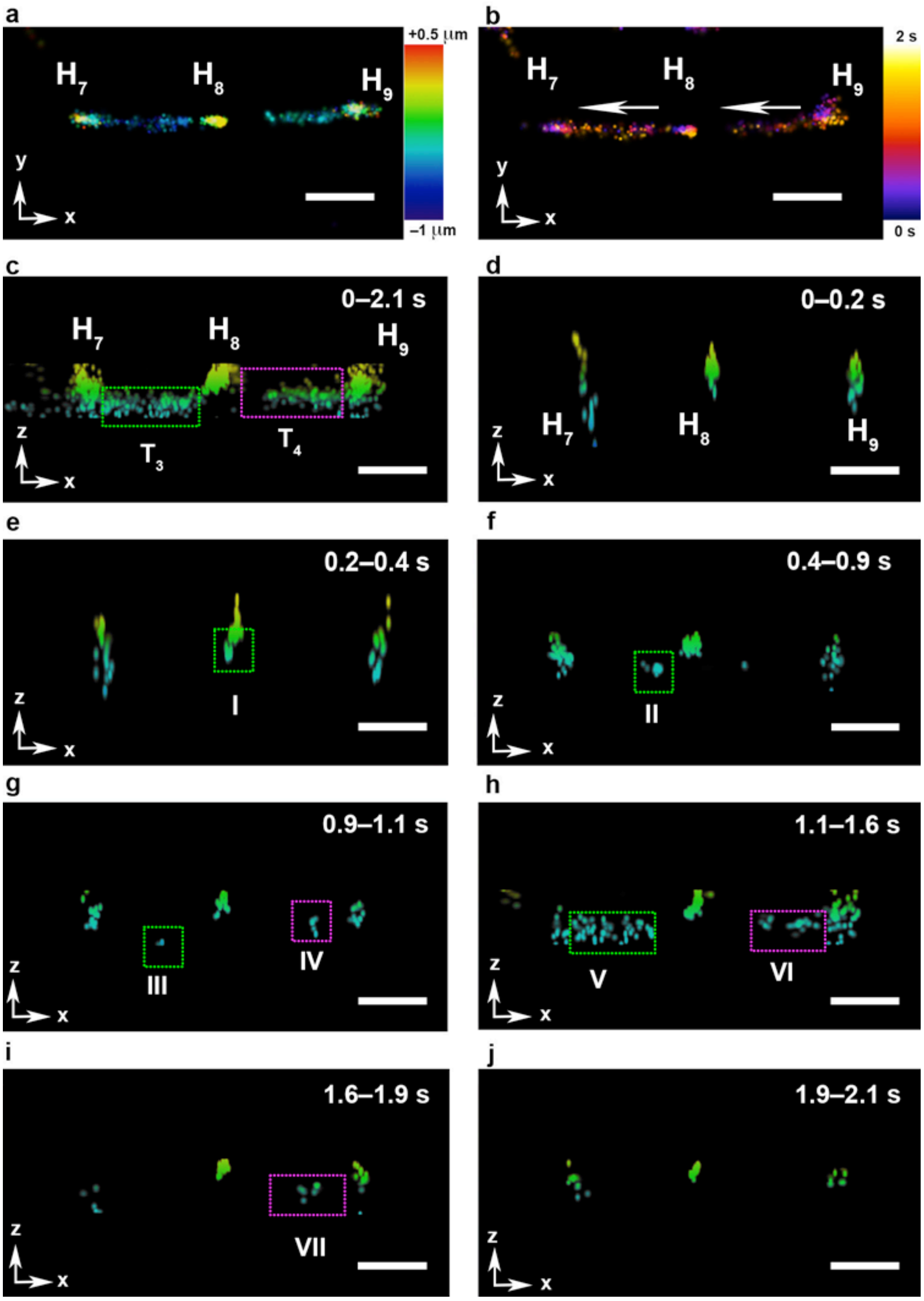

Supplementary Figure 19 | Additional example of 3D SMLM of acidic vesicles hopping between three hotspots ($H_{7\text{-}9}$). a, Formation of two diffusion tracks (T_3 and T_4) between $H_{7\text{-}9}$ shown in the lateral axis (x-y) with a color-coded depth of 1.5 μm. **b**, 2D depiction of the same time acquisition as in panel **a** color coded in time (2.1 s). The directionality of the tracks is indicated by the arrows. **c**, 3D

reconstruction of panel **a** projected on the axial plane (z-x), displaying two tracks (T_3 and T_4) between ($H_{7\text{-}9}$) on different x-y planes. **d-i**, Sequential reconstruction of the diffusion trails shown in panels **a-c**: Box **I** = budding of an acidic vesicle out of H_8, boxes **II–III** = vesicle entering track T_3; box **IV** = emergence of an additional acidic vesicle from H_9; boxes **V–VII** = unresolved vesicle moving fast along the tracks. **j**, End of the diffusion cycle. Scale bars = 1.5 μm.

Supplementary Table 1. Comparison of irradiation intensities in selected typical STORM experiments and in this work. PA = Photoactivation.

PA wavelength, intensity	PA pulses, duration	Imaging wavelength, intensity	reference
**405 nm, 2.6 W cm^{-2}**	**every 10 min, 20 ms**	**561 nm, 0.25 kW cm^{-2}**	**This work**
405 nm, 1 kW cm^{-2}	continuous illumination	561 nm, 5-20 kW cm^{-2}	*Nat. Methods* **8**, 499 (2011)
405 nm, 3 W cm^{-2}	continuous illumination	561 nm, 10 kW cm^{-2}	*Proc. Natl. Acad. Sci. U. S. A.* **109**, 13978 (2012)
407 nm, 50 W cm^{-2}	every 1 s, 100 μs	561 nm, 1.7 kW cm^{-2}	*Nat. Methods **13,*** 985 (2016)
---	---	647 nm, 1.5-10 kW cm^{-2}	*Nat. Biotechol.* **35**, 773 (2017)
---	---	647 nm, 2-4 kW cm^{-2}	*Nat. Commun.* ***9,*** 930 (2018)

Supplementary Methods

2-Amino-3',6'-bis(diethylamino)spiro[isoindoline-1,9'-xanthen]-3-one (3)

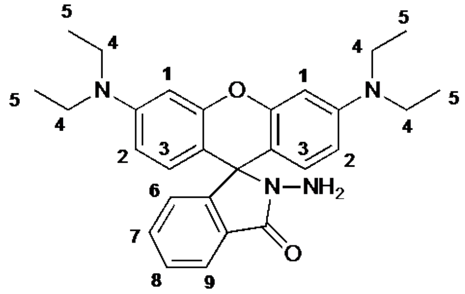

Rhodamine B (2 g, 4.17 mmol) was dissolved in methanol (75 mL) and hydrazine hydrate 50% (35 mL) was added. The mixture was stirred at 25 ^{o}C for 2 h. The color of the reaction changed from dark red to pale orange and a precipitate formed. The organic solvent was evaporated under reduced pressure and diluted

with HCl (1 M) until complete dissolution of the compound (50 mL). Aqueous NaOH (1 M) was subsequently added until a precipitate formed. The precipitate was filtered and dried in a desiccator for 18 h to give compound **3**, as a pale pink solid (1.6 g, 3.5 mmol, 84% yield). ^{1}H NMR (400 MHz, CD_3OD) δ = 7.88 (dd, J = 6.3, 1.5 Hz, 1H, H9), 7.51 (td, J = 6.7, 1.5 Hz, 1H, H7), 7.47 (td, J = 6.3, 1.3 Hz, 1H, H8), 7.07 (dd, J = 6.7, 1.6 Hz, 1 H, H6), 6.51–6.44 (m, 2H, H3), 6.41 (m, overlapping signals, 4H, H1, H2), 3.40 (q, J = 7.1 Hz, 8H, H4), 1.18 (t, J = 7.1 Hz, 12H, H5). ^{13}C NMR (400 MHz, CD_3OD) δ = 155.26, 150.39, 134.05, 129.43, 128.78, 124.96, 123.60, 109.52, 105.45, 99.39, 49.50, 49.28, 49.07, 48.85, 45.40, 12.86. HRMS (ESI) calcd for $[C_{28}H_{33}N_4O_2]^+$: 457.2598, found: 457.2610.

General procedure for the synthesis of PFF-1 and 2.

Intermediate **3** (1 equiv.) was dissolved in ethanol (2 mL) followed by the respective aldehyde (1.1–1.4 equiv.) and 2 drops of trifluoroacetic acid. A precipitate formed and the reaction was stirred at 35 ^{o}C for 20 min, filtered, washed with ethanol and dried under vacuum to give the desired final compound as a white or pale orange solid.

(*E*)-3',6'-bis(Diethylamino)-2-((pyridin-2-ylmethylene)amino)spiro[isoindoline-1,9'-xanthen]-3-one (PFF-1).

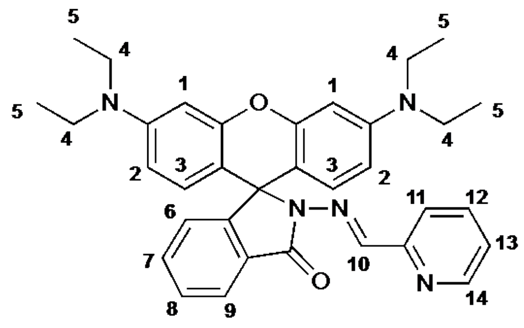

Following the general procedure, compound **3** (200 mg, 0.43 mmol) and picolinaldehyde (64 mg, 0.602 mmol) gave compound **PFF-1** (203 mg, 0.37 mmol, 86%) as an off-white solid. ^{1}H NMR (400 MHz, $CDCl_3$) δ = 8.49 (d, J = 4.9

Hz, 1H, H14), 8.41 (s, 1H, H10), 8.03 (m, overlapping signal, 2H, H9 and H11), 7.62 (td, *J* = 7.8, 1.7 Hz, 1H, H12), 7.51 (td, *J* = 7.4, 1.5 Hz, 1H, H7), 7.47 (td, *J* = 7.3, 1.3 Hz, 1H, H8), 7.18–7.10 (m, overlapping signals, 2H, H6 and H13), 6.57 (d, *J* = 8.8, 2H, H3), 6.47 (d, *J* = 2.6 Hz, 2H, H1), 6.26 (dd, *J* = 8.9, 2.6 Hz, 2H, H2), 3.3 (q, *J* = 7.1, 8H, H4), 1.17 (t, *J* = 7.0 Hz, 12H, H5). ^{13}C NMR (400 MHz, $CDCl_3$) δ = 165.47, 154.64, 152.89, 152.53, 148.98, 148.94, 145.91, 136.07, 133.71, 128.23, 128.01, 127.58, 123.71, 123.61, 123.54, 120.60, 108.03, 105.58, 98.30, 65.83, 44.31, 12.63. HRMS (ESI) calcd for $[C_{34}H_{36}N_5O_2]^+$: 457.2864, found: 457.2876.

(*E*)-2-(Benzylideneamino)-3',6'-bis(diethylamino)spiro[isoindoline-1,9'-xanthen]-3-one (2).

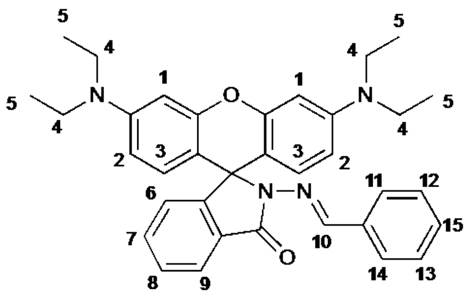

Following the general procedure**,** compound **3** (200 mg, 0.43 mmol) and benzaldehyde (63 mg, 0.602 mmol) gave product **2** (187 mg, 0.34 mmol, 78%) as an off-white solid. ^{1}H NMR (400 MHz, $CDCl_3$) δ = 8.70 (s, 1H, H10), 8.01 (dd, *J* = 6.5, 2.1 Hz, 1H, H9), 7.62–7.55 (m, overlapping signals, 2H, H11 and H14), 7.55–7.45 (m, overlapping signals, 2H, H7 and H8), 7.29 (m, overlapping signals, 3H, H12, H13 and H15), 7.19–7.10 (dd, *J* = 6.5, 2.2 Hz, H1, H6), 6.55 (d, *J* = 8.8 Hz, 2H, H3), 6.46 (d, *J* = 2.6 Hz, 2H, H1), 6.26 (dd, *J* = 8.9, 2.6 Hz, 2H, H2), 3.34 (q, *J* = 7.1 Hz, 8H, H4), 1.18 (t, *J* = 7.0 Hz, 12H, H5).^{13}C NMR (400 MHz, $CDCl_3$) δ = 164.93, 153.13, 151.80, 148.91, 147.26, 135.44, 133.28, 129.56, 129.38, 128.23, 128.03, 127.52, 123.85, 123.34, 108.00, 106.11, 97.87, 77.21, 65.99, 44.32, 12.62. HRMS (ESI) calcd for $[C_{35}H_{37}N_4O_2]^+$: 545.2911, found: 545.2900.

N_3,N_3,N_7,N_7,5,5-hexamethyl-3'*H*,5*H*-spiro[dibenzo[*b*,*e*]siline-10,1'-isobenzofuran]-3,7-diamine, (HMSiR, 4)

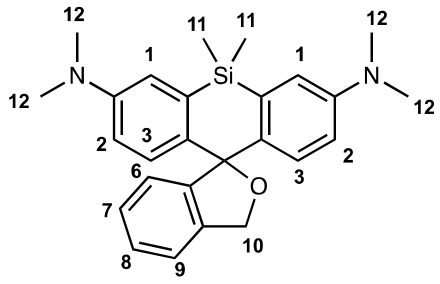

3,3'-(Dimethylsilanediyl)bis(4-bromo-*N*-,*N*-dimethylaniline[1] (100 mg, 0.219 mmol) was carefully dried under vacuum, dissolved in 5 mL of dry THF, and cooled to –78 °C. *t*BuLi in hexanes (1.7 M, 0.58 mL, 0.464 mmol) was added and the reaction was stirred for 30 min at –78 ^{o}C. The reaction was warmed to –10 ^{o}C, $MgBr_2OEt_2$ (114 mg, 0.481 mmol) was added, and the mixture was stirred for 30 min at –10 °C. A solution of phtalide (35 mg, 0.263 mmol) in THF (5 mL) was slowly added over 30 min. The mixture was stirred for 18 h at 25 °C. Upon completion of the reaction, THF was evaporated under reduced pressure, and the residue was dissolved in EtOAc, washed with a saturated NH_4Cl solution, $NaHCO_3$, and brine. The organic phase was dried with $MgSO_4$. Flash column chromatography (SiO_2 Petrol Ether:CH_2Cl_2:EtOAc 75:5:20) afforded the desired product as pale green crystals of HMSiR (27 mg, 30%). ^{1}H NMR (400 MHz, $CDCl_3$) δ = 7.37–7.29 (m, 2H, H7, H8), 7.32–7.22 (m, 1H, H9, overlapping signals), 7.12–7.07 (d, J = 7.2 Hz, 1H, H6), 7.05–6.96(m, 4H, H1, H3), 6.65 (dd, J = 8.9, 2.9 Hz, 2H, H2), 5.26 (s, 2H, H10), 2.97 (s, 12H, H12), 0.65 (s, 3H, H11a), 0.58 (s, 3H, H11b). ^{13}C NMR (400 MHz, $CDCl_3$) δ = 148.70, 146.11, 139.76, 138.43, 135.54, 128.55, 127.23, 124.60, 121.00, 116.76, 113.80, 92.50, 76.71, 72.28, 40.55, 0.63, –1.24. HRMS (ESI) calcd for $[C_{26}H_{31}N_2OSi]^+$: 415.2200, found: 415.2207.

Ethyl (*E*)-2-(((3',6'-bis(diethylamino)-3-oxospiro[isoindoline-1,9'-xanthen]-2-yl)imino)methyl)isonicotinate (5)

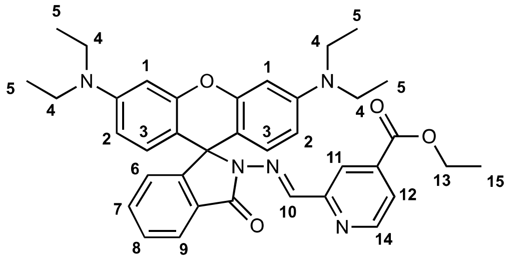

Following the general procedure, compound **3** (254 mg, 0.55 mmol) and ethyl 2-formylisonicotinate (100 mg, 0.55 mmol)[2] gave compound **5** (300 mg, 0.48 mmol, 87%) as an off-white solid. ^{1}H NMR (400 MHz, $CDCl_3$) δ = 8.62 (dd, J = 5.0, 0.9 Hz, 1 H, H14), 8.50 (s, H1, H11), 8.44 (s, 1H, H10), 8.05 (d, J = 6.6 Hz, 1H, H9), 7.71 (dd, J = 5.0, 1.6 Hz, 1H, H12), 7.54 (td, J = 7.4, 1.5 Hz, 1H, H7), 7.49 (td, J = 7.4, 1.3 Hz, 1H, H8), 7.15 (dd, J = 7.1, 1.2 Hz, 1H, H8), 6.66–6.49 (m, 4H, H1, H3), 6.31 (dd, J = 9.1, 2.6 Hz, 2H, H2), 4.43 (q, J = 7.1 Hz, 2H, H13), 3.34 (q, J = 7.0 Hz, 9H, H4), 1.45 (t, J = 7.1 Hz, 3H, H15), 1.17 (t, J = 7.0 Hz, 13H, H5). ^{13}C NMR (400 MHz, $CDCl_3$) δ = 165.44, 165.04, 155.59, 152.89, 152.27, 149.60, 148.50, 145.01, 138.15, 133.92, 128.43, 127.93, 127.66, 123.77, 122.56, 119.92, 108.59, 106.21, 99.12, 76.83, 65.86, 61.80, 44.81, 14.25, 12.47. HRMS (ESI) calcd for $[C_{37}H_{38}N_5O_4]^+$: 618.3075, found: 618.3075.

2,5-Dioxopyrrolidin-1-yl (*E*)-2-(((3',6'-bis(diethylamino)-3-oxospiro[isoindoline-1,9'-xanthen]-2-yl)imino)methyl)isonicotinate (6)

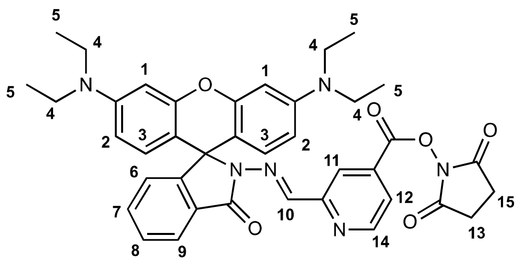

Ethyl ester rhodamine **5** (130 mg, 0.21 mmol) was diluted in MeOH (3 mL) and treated with LiOH (70 mg, 1.62 mmol) at 60 °C for 2 h. The solvent was evaporated and dissolved in CH_2Cl_2 (5 mL). The mixture was acidified with aqueous HCl (1 M) and extracted with CH_2Cl_2. The organic layer was washed with brine and dried over $MgSO_4$. The solvents were removed under reduced pressure and the crude was crushed with petrol ether to give (*E*)-2-(((3',6'-bis(diethylamino)-3-oxospiro[isoindoline-1,9'-

xanthen]-2-yl)imino)methyl)isonicotinic acid as an off-white solid (95 mg, 0.16 mmol, 76%), which was used without further purification. The acid (50 mg, 0.084 mmol) and *N*-hydroxysuccinimide (NHS, 9.6 mg, 0.084 mmol) were dissolved in anhydrous $CNCH_3$ (1 mL). *N*,*N'*-Dicyclohexylcarbodiimide (DCC, 17.5, 0.084 mmol) was added and the mixture was stirred for 1 h. After completion of the reaction, the mixture was filtered through a silica plug and washed with $CNCH_3$. The solvents were removed under reduced pressure to give the desired product **6** as a off-white solid (30 mg, 0.043 mmol, 51%). ^{1}H NMR (400 MHz, $CNCD_3$) δ = 8.74 (dd, *J* = 5.1, 0.9 Hz, 1H, H14), 8.42 (s, 1H, H10), 8.33 (dd, *J* = 1.7, 0.9 Hz, 1H, H11), 7.98 (dd, *J* = 7.5, 2.1 Hz, 1H, H9), 7.82 (dd, *J* = 5.1, 1.7 Hz, 1H, H12), 7.64 (td, *J* = 7.5, 1.4 Hz, 2H, H7), 7.58 (td, *J* = 7.4, 1.2 Hz, 2H, H8), 7.11 (dt, *J* = 7.7, 0.9 Hz, 1H, H6), 6.54 (d, *J* = 8.8 Hz, 2H, H3), 6.49 (d, *J* = 2.6 Hz, 2H, H1), 6.36 (dd, *J* = 8.9, 2.6 Hz, 2H, H2), 3.36 (q, *J* = 7.0 Hz, 8H, H4), 2.89 (s, 4H, H13, H15), 1.12 (t, *J* = 7.0 Hz, 12H, H5). ^{13}C NMR (400 MHz, $CNCD_3$) δ = 169.70, 164.80, 160.98, 155.68, 152.92, 152.27, 151.10, 149.26, 143.37, 134.34, 133.23, 128.82, 128.01, 127.70, 123.58, 123.39, 122.68, 118.54, 117.31, 108.25, 104.93, 97.51, 43.99, 25.52, 11.77. HRMS (ESI) calcd for $[C_{39}H_{39}N_6O_6]^+$: 687.2926, found: $[C_{35}H_{35}N_5O_4]^+$ = $[M-C_4H_4NO_2]^+$ = 589.2918.

(*E*)-(3-(2-(((3',6'-bis(diethylamino)-3-oxospiro[isoindoline-1,9'-xanthen]-2-yl)imino)methyl)isonicotinamido)propyl)triphenylphosphonium (MitoPFF-1)

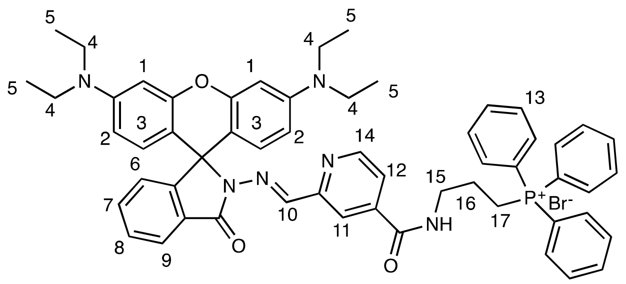

Compound **6** (50 mg, 0.072 mmol) and (3-aminopropyl)triphenylphosphonium (42 mg, 0.087 mmol) were dissolved in anhydrous DMF (3 mL) and triethylamine (50 μL) was added under inert atmosphere (N_2). The reaction was stirred for 36 h at 25 ^oC. Upon completion of the reaction, DMF was removed by rotary evaporation and the crude was diluted with CH_2Cl_2 and extracted with brine. The organic layer was dried with $MgSO_4$ and concentrated. The crude was crushed with petrol ether:ethylacetate (3:1) and sonicated to obtain a pale green precipitate, which was treated with acetone to yield the desired compound **MitoPFF-1**, as a white solid (25 mg, 36% yield). ^1H NMR (400 MHz, $CDCl_3$) δ = 9.92 (t, *J* = 5.8 Hz, 1H, NH), 8.62 (d, *J* = 5.1 Hz, 1H, H14), 8.48 (s, 1H, H10), 8.18 (d, *J* = 5.2 Hz, 1H, H9), 8.08 (s, 1H, H11), 8.02 (d, *J* = 7.5 Hz, 1H, H12), 7.85 – 7.57 (m, 15H, H13), 7.46 (tq, *J* = 14.7, 7.3 Hz, 2H, H7 and H8), 7.10 (d, *J* = 7.4 Hz, 1H, H6), 6.59 (d, *J* = 8.8 Hz, 2H, H3), 6.47 (d, *J* = 2.5 Hz, 2H, H1), 6.23 (dd, *J* = 8.7, 2.3 Hz, 2H, H2), 3.98 – 3.82 (m, 2H, H15), 3.74 (d, *J* = 6.0 Hz, 2H, H17), 3.32 (q, *J* = 7.0 Hz, 8H, H4), 2.01 (d, *J* = 16.1 Hz, 2H, H16, signal overlap with Acetone), 1.15 (t, *J* = 7.0 Hz, 12H, H5). ^{13}C NMR (400 MHz, $CDCl_3$) δ = 165.80, 154.76, 152.58, 149.95, 149.06, 144.89, 141.47, 135.18, 135.15, 133.81, 133.48, 133.38, 130.62, 130.50, 128.09, 127.36, 123.65, 123.53, 121.66, 119.51, 118.64, 117.79, 108.14, 105.16, 98.50, 44.31, 22.17, 20.80, 20.28, 12.65. HRMS (ESI) calcd for $[C_{56}H_{56}N_6O_3P]^+$: 891.4146, found: 891.4127.

(E)-4-(4-(dibutylamino)styryl)-1-(3-(triethylammonio)propyl)pyridin-1-ium (FM1-43)[3]

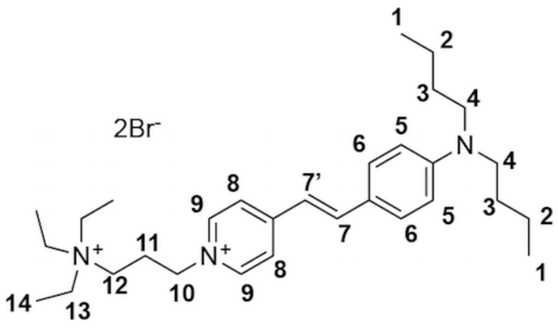

(E)-*N*,*N*-dibutyl-4-(2-(pyridin-4-yl)vinyl)aniline[4] (1.5 g, 4.9 mmol) and 3-bromo-*N,N,N*-triethylpropan-1-ammonium bromide (1.3 g, 4.2 mmol) were mixed in dry CH_3CN (60 mL) in N_2 atmosphere. The mixture was maintained at reflux for 24 h. The solvents were evaporated and the residue was recrystallized from acetone to give FM1-43 (1,6 g, 60%) deep red solid. ^{1}H NMR (400 MHz, CD_3CN) δ = 8.67 (d, J = 7.0 Hz, 2H, H9), 7.61 (d, J = 7.0 Hz, 2H, H8), 7.52 (d, J = 16 Hz, 1H, H7'), 7.27 (d, J = 9.0 Hz, 2H, H6), 6.75 (d, J = 16.0 Hz, 1H, H7), 6.45 (d, J = 9 Hz, 1 H, H5), 4.49–4.32 (m, 2 H, H10), 3.21–3.13 (m, 2H, H12), 3.13–3.04 (m, 4H, H4), 3.00 (q, J = 7.2 Hz, 6H, H1), 2.14 (q, J = 8.1 Hz, 2H, H11), 1.30 (dq, J = 9.1, 2.5 Hz, 4H, H3), 1.14–1.03 (m, 4H, H2), 1.00 (q, J = 7.2 Hz, 9H, H14), 0.67 (t, J = 7.4 Hz, 6H, H13) ppm. ^{13}C NMR (400 MHz, $CNCD_3$) δ = 155.05, 150.65, 143.57, 143.10, 130.77, 122.30, 121.81, 116.09, 111.73, 55.62, 53.23, 50.30, 29.11, 24.04, 19.87, 13.24, 7.13 ppm. HRMS (ESI-QTOF) calcd for $[C_{30}H_{49}N_3]^{+2}$: 225.6958, found: 225.6961.

(2a*R*,4*S*,4a*S*,6*R*,9*S*,11*S*,12*S*,12a*R*,12b*S*)-12b-acetoxy-9-((3-(2-((*E*)-((3',6'-bis(diethylamino)-3-oxospiro[isoindoline-1,9'-xanthen]-2-yl)imino)methyl)isonicotinamido)-2-hydroxy-3-phenylpropanoyl)oxy)-4,6,11-trihydroxy-4a,8,13,13-tetramethyl-5-oxo-2a,3,4,4a,5,6,9,10,11,12,12a,12b-dodecahydro-1*H*-[7,11]methanocyclodeca[3,4]benzo[1,2-*b*]oxet-12-yl benzoate (TaxolPFF-1)

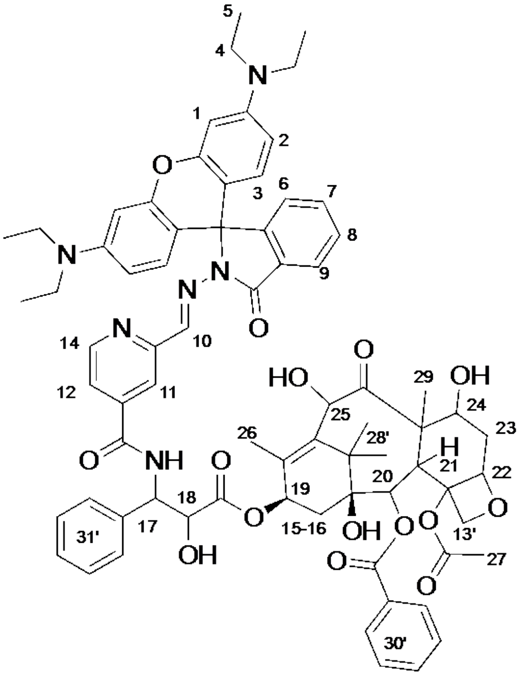

Docetaxel (40 mg, 0.049 mmol) was dissolved in formic acid (1 mL) while cooling with an ice bath. The solution was allowed to stir for 40 min at 25 ^{o}C and monitored by LC-MS. Formic acid was removed under high vacuum and the crude was extracted with sodium bicarbonate and EtOAc, dried and evaporated. The crude (30 mg, 0.042 mmol) was dissolved in anhydrous DMF (0.5 mL) and treated with anhydrous Et_3N (0.042 mmol). A solution of compound **6** (57 mg, 0.084 mmol) in DMF (0.5 mL) was slowly added at 0 ^{o}C and allowed to warm to room temperature. The reaction was monitored by LC-MS. Upon completion of the reaction, the solution was extracted with EtOAC and washed with abundant brine, dried over $MgSO_4$ and the organic solvents were evaporated. The final product was purified by reverse-

phase HPLC (H_2O: $CNCH_3$, 50% → 100 %) and obtained as a white solid (10 mg, 0.0078 mmol, 19% yield). ^1H NMR (500 MHz, $CNCD_3$) δ = 8.56 (dd, J = 5.1, 0.8 Hz, 1H, H14), 8.33 (s, 1H, H10), 8.17 (dd, J = 1.7, 0.9 Hz, 1H, H9), 8.13–8.11 (m, 1H, H30’), 8.10 (dd, J = 2.0, 1.4 Hz, 1H, H11), 7.96 (ddd, J = 7.6, 1.3, 0.8 Hz, 1H, H30’), 7.67–7.61 (m, 2H, H30’ overlapping signals), 7.61–7.55 (m, 4H, H7, H8, H12, H30’ overlapping signals), 7.50 (ddd, J = 7.9, 1.4, 0.7 Hz, 2H, H31’), 7.47–7.43 (m, 2H, H31’), 7.35–7.31 (m, 1H, H31’), 7.10 (dt, J = 7.7, 0.9 Hz, 1H, H6), 6.35–6.46 (m, 4H, H1, H3), 6.35 (dt, J = 8.9, 2.9 Hz, 2H, H2), 6.14 (td, J = 9.0, 1.7 Hz, 1H, H24), 5.24 (dd, J = 8.8, 4.3 Hz, 1H, H17), 5.59 (d, J = 7.1 Hz, 1H, H13’), 5.23–5.17 (m, 1H, H25), 4.96 (dd, J = 9.7, 2.2 Hz, 1H, H19), 4.75 (d, J = 4.2 Hz, 1H, H18), 4.37 (s, 1H, NH), 3.84 (d, J = 7.1 Hz, 1H, H13), 3.38–3.31 (m, 8H, H4), 3.29–3.25 (m, 1H, H20), 2.42 (m, 1H, H16), 2.37 (s, 3H, H27), 2.26 (m, 2H, H23, overlapping signals), 1.92 (s, 3H, H29), 1.80–1.73 (m, 1H, H15), 1.68 (s, 3H, H28’), 1.58–1.51 (m, 1H, H 22), 1.39–1.33 (m, 1H, H21), 1.15–1.08 (m, 18H, H5, H28’, overlapping signals), 1.06 (s, 3H, H26). ^{13}C NMR (500 MHz, $CNCD_3$) δ = 210.80, 172.43, 170.58, 165.90, 165.37, 165.16, 154.52, 152.88, 152.45, 150.20, 149.28, 144.55, 142.14, 138.63, 137.65, 136.49, 134.35, 133.46, 130.14, 129.96, 128.81, 128.68, 128.61, 127.87, 127.67, 127.36, 123.52, 123.37, 121.77, 116.93, 108.29, 108.23, 97.56, 84.05, 80.83, 77.90, 76.11, 74.89, 74.36, 73.65, 71.39, 71.37, 57.56, 56.16, 46.47, 43.98, 43.02, 36.54, 35.93, 26.26, 22.21, 20.38, 13.81, 11.80, 11.78, 9.50. HRMS (ESI-QTOF) calcd for $[C_{73}H_{79}N_6O_{15}]^+$: 1279.5598, found: 1279.5593.

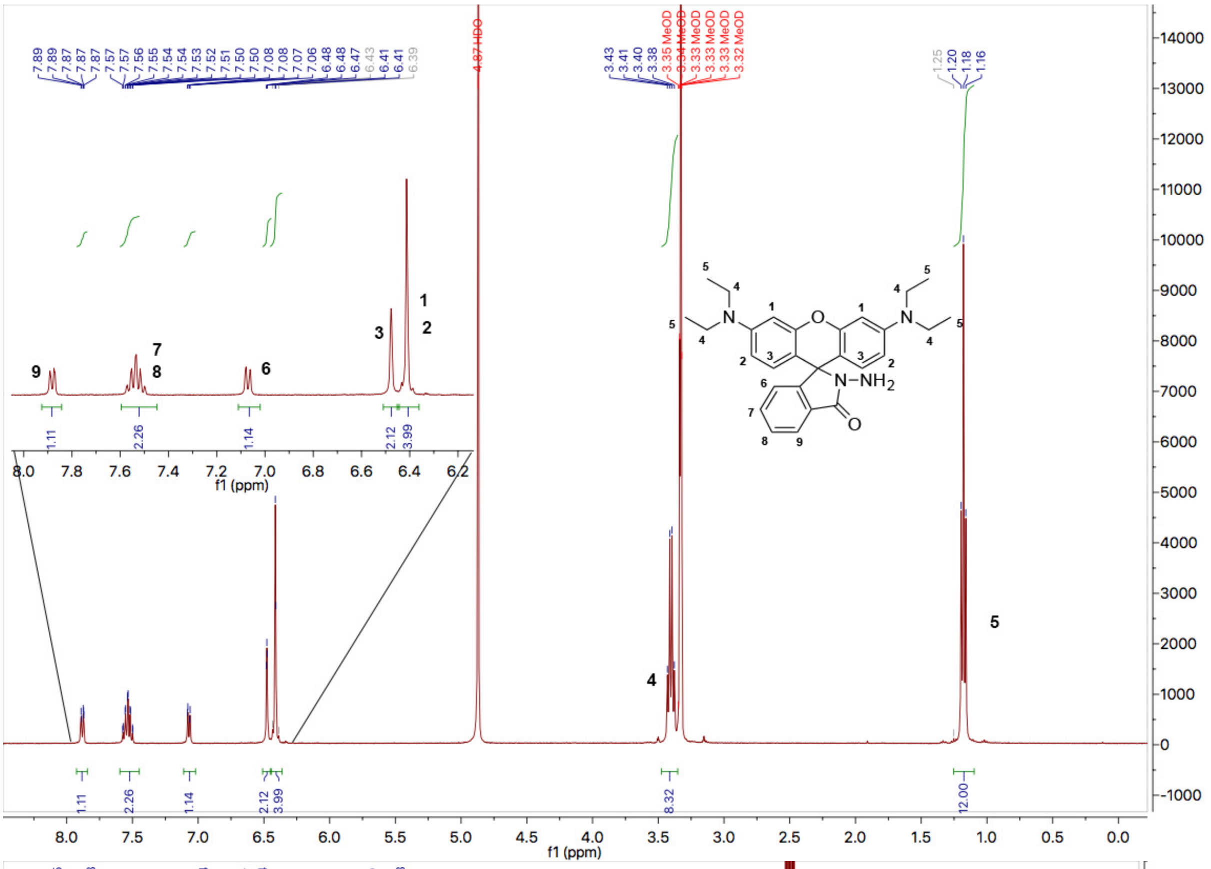

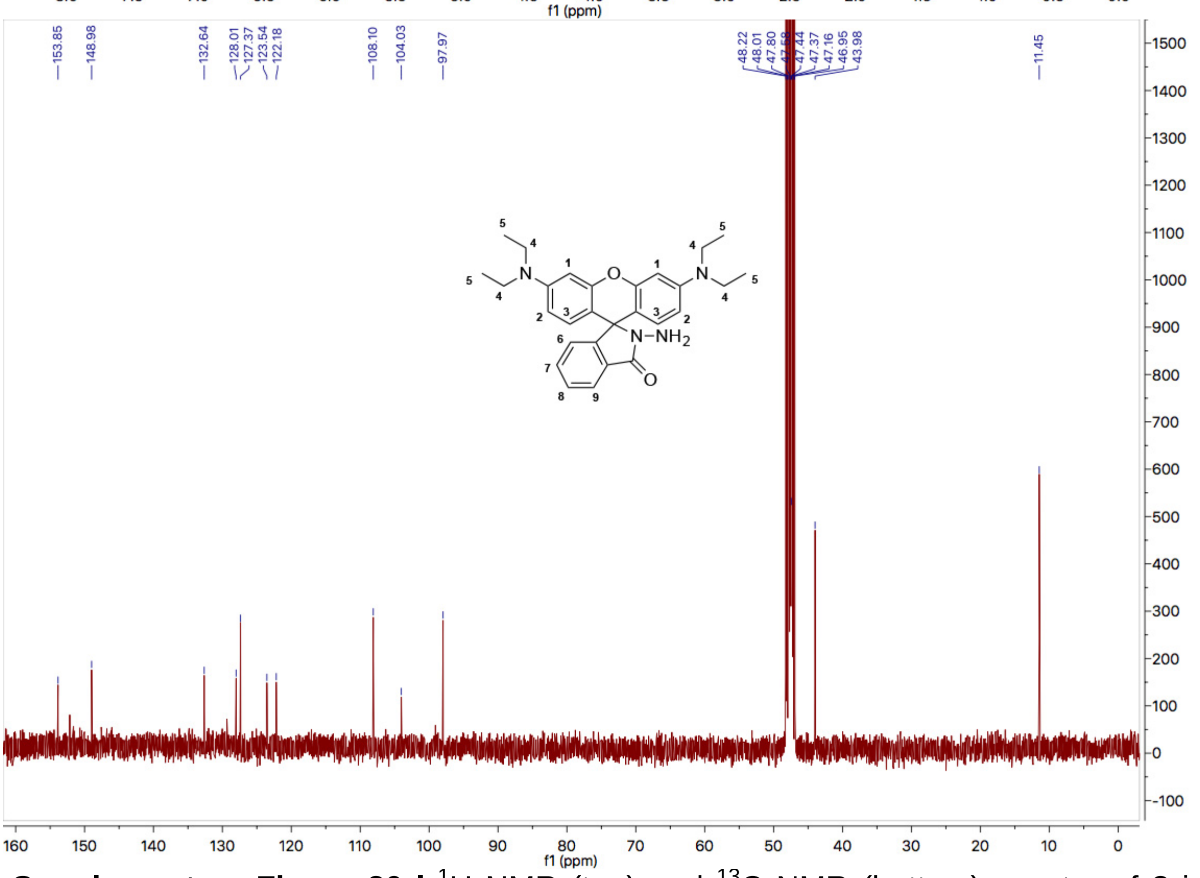

Supplementary Figure 20 | ^{1}H NMR (top) and ^{13}C NMR (bottom) spectra of **3** in CD_3OD.

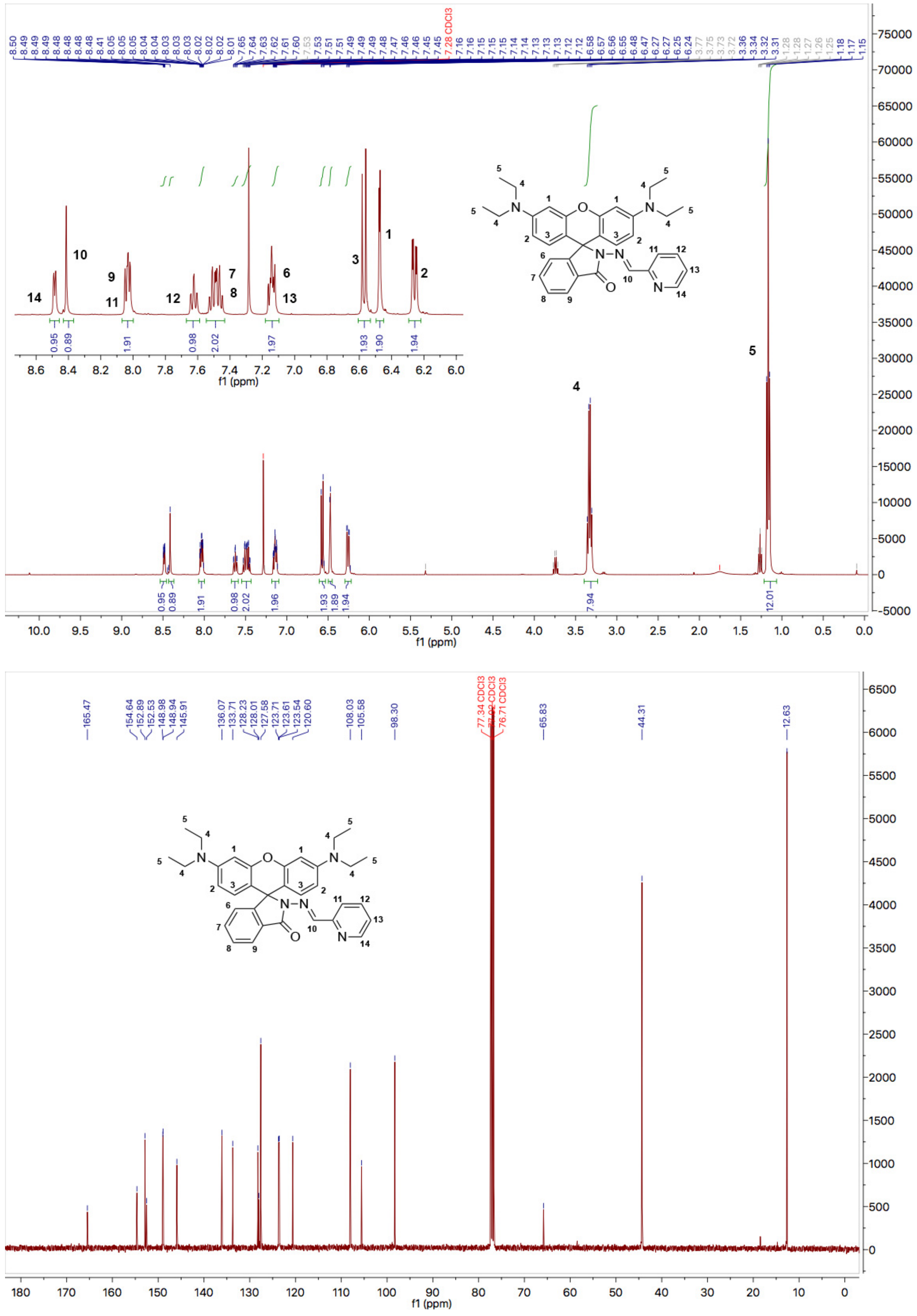

Supplementary Figure 21 | ^{1}H NMR (top) and ^{13}C NMR (bottom) spectra of PFF-**1** in $CDCl_3$.

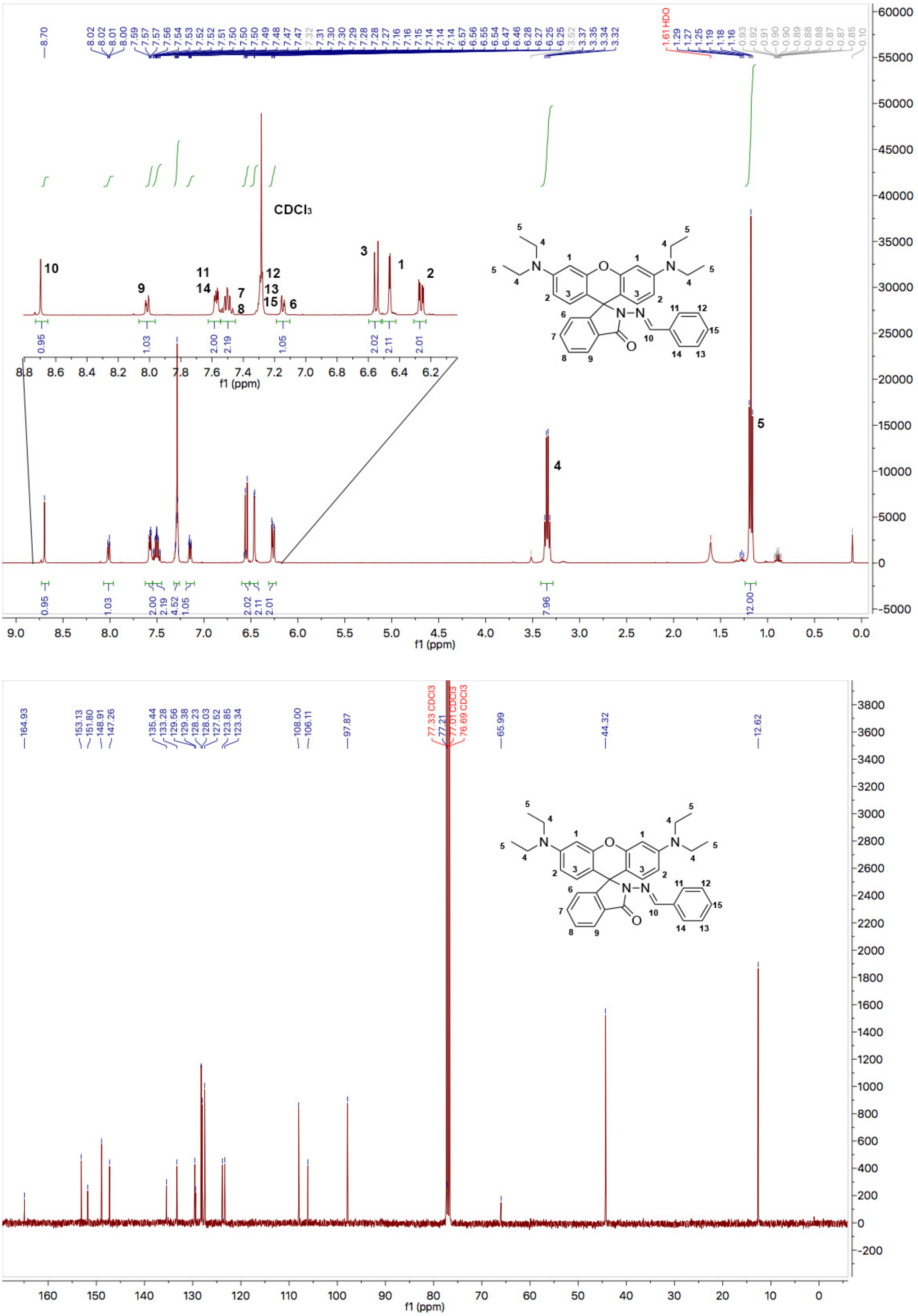

Supplementary Figure 22 | ^{1}H NMR (top) and ^{13}C NMR (bottom) spectra of **2** in $CDCl_3$.

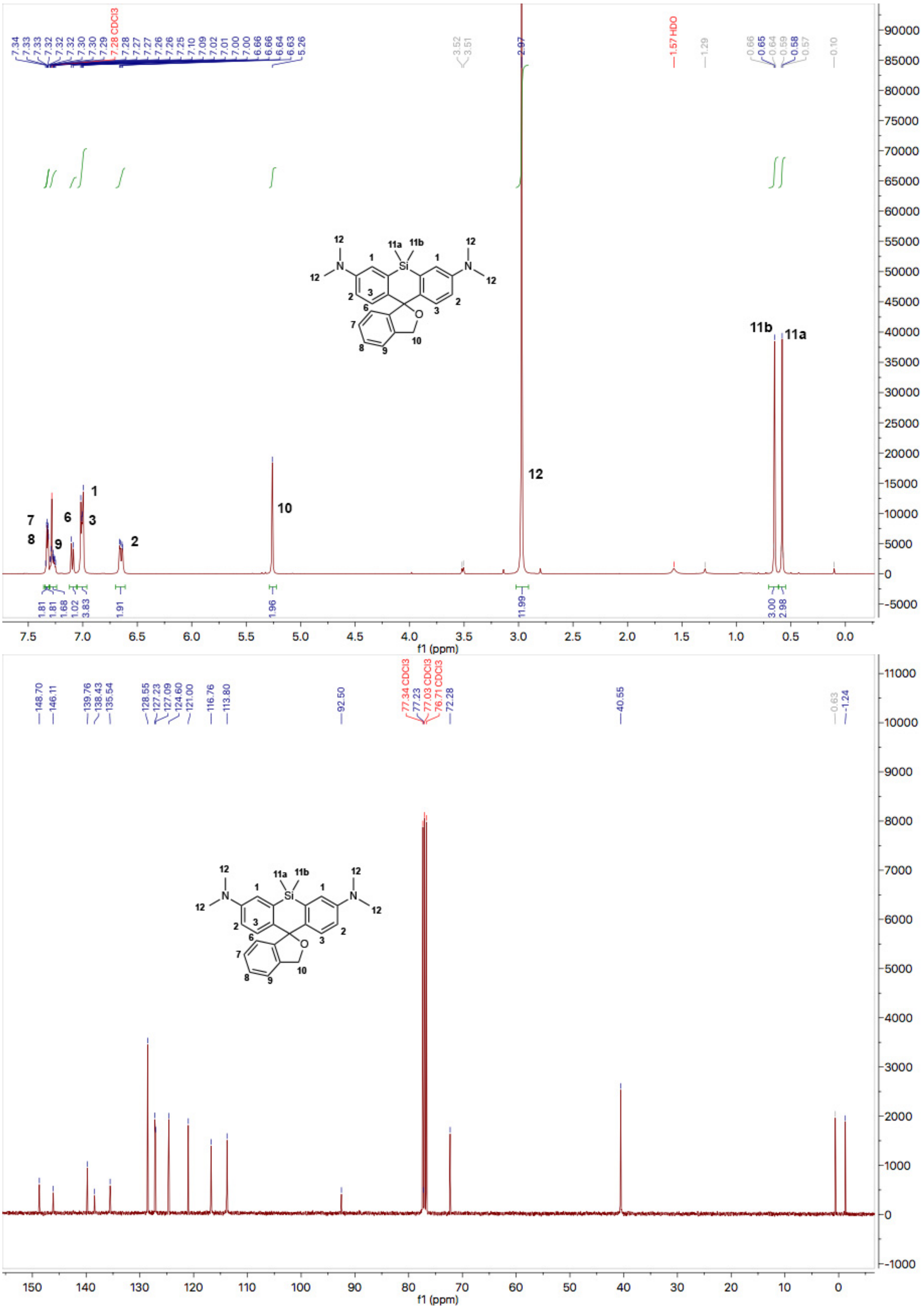

Supplementary Figure 23 | ^{1}H NMR (top) and ^{13}C NMR (bottom) spectra of HMSiR in $CDCl_3$.

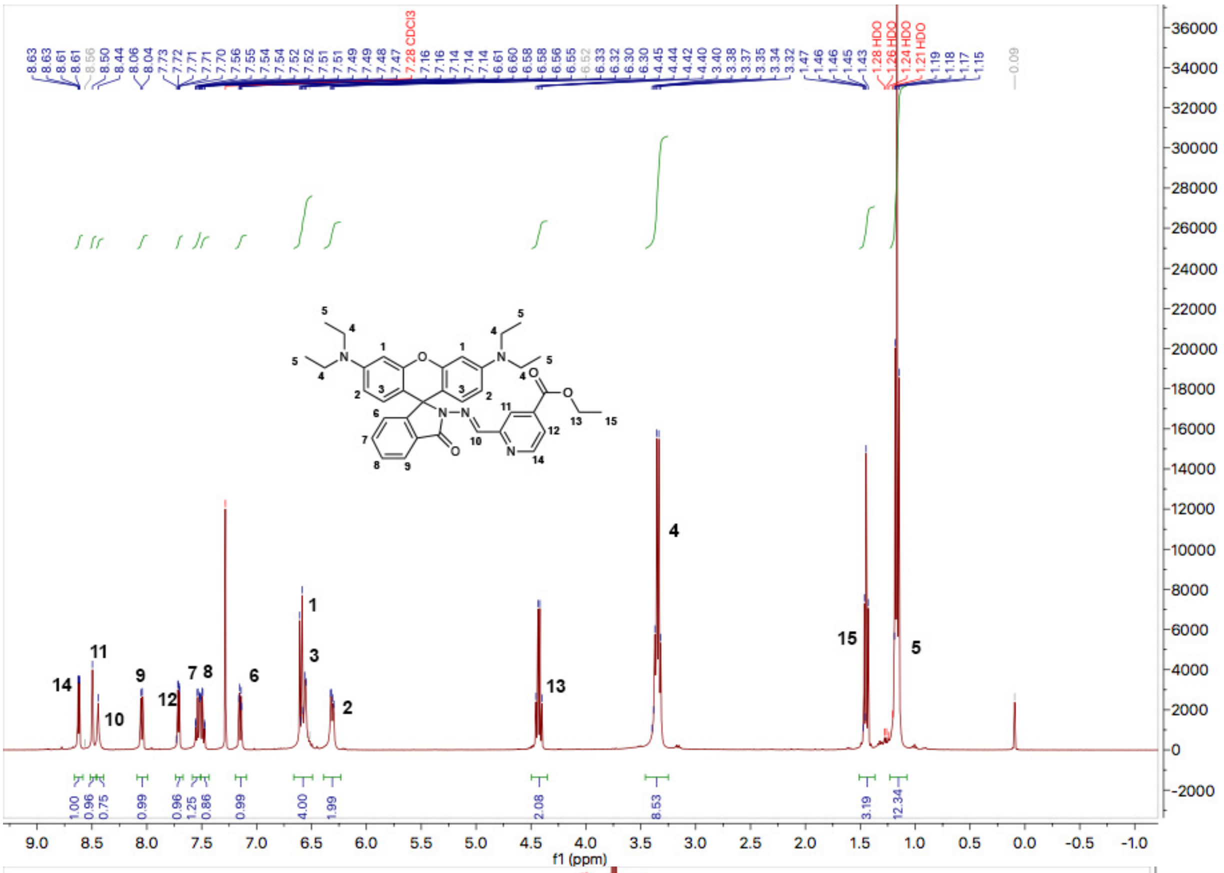

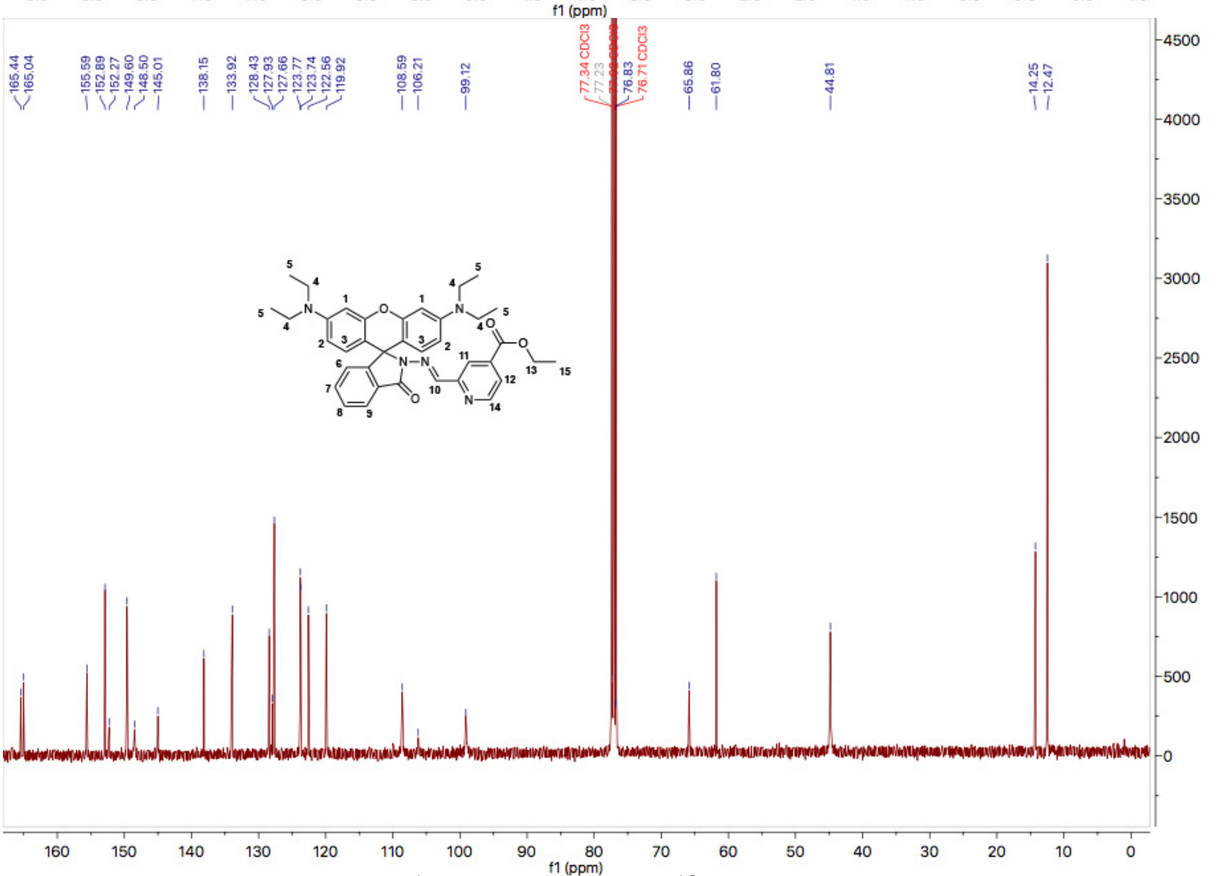

Supplementary Figure 24 | ^{1}H NMR (top) and ^{13}C NMR (bottom) spectra of **5** in $CDCl_3$.

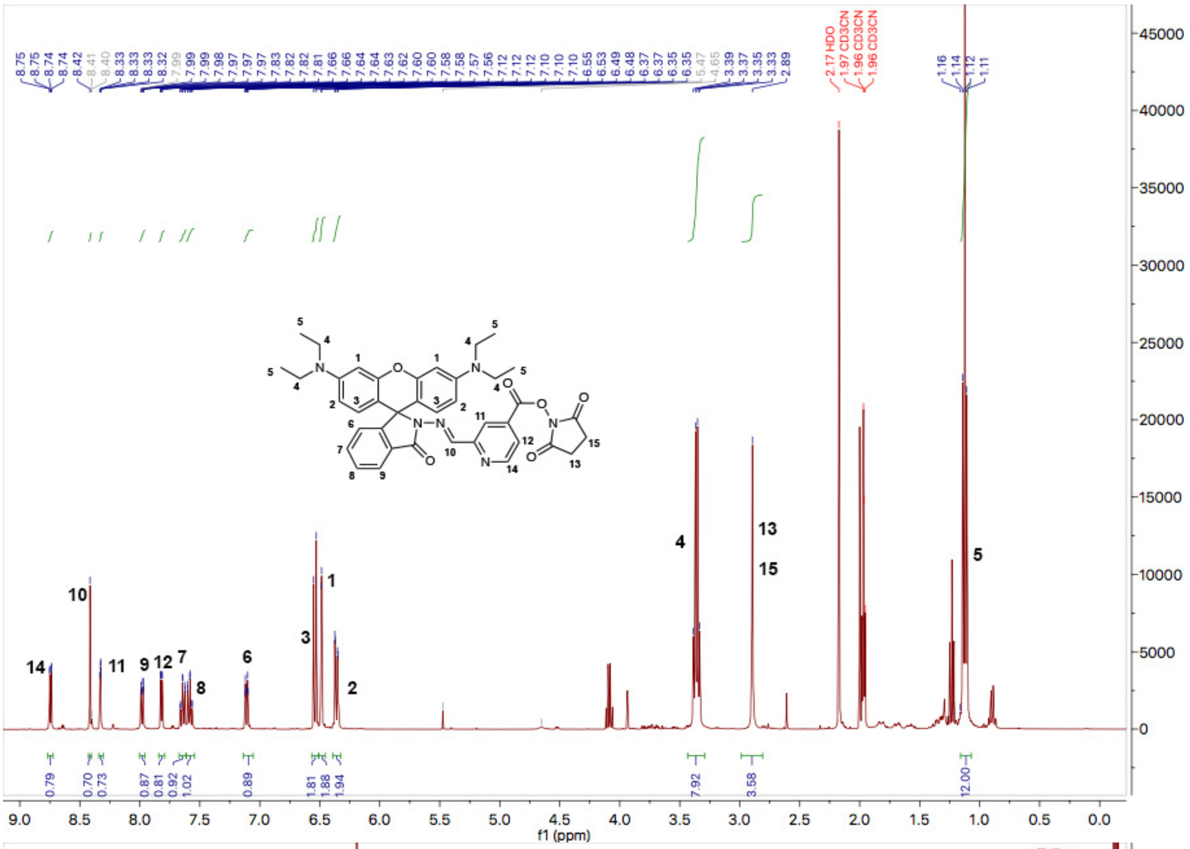

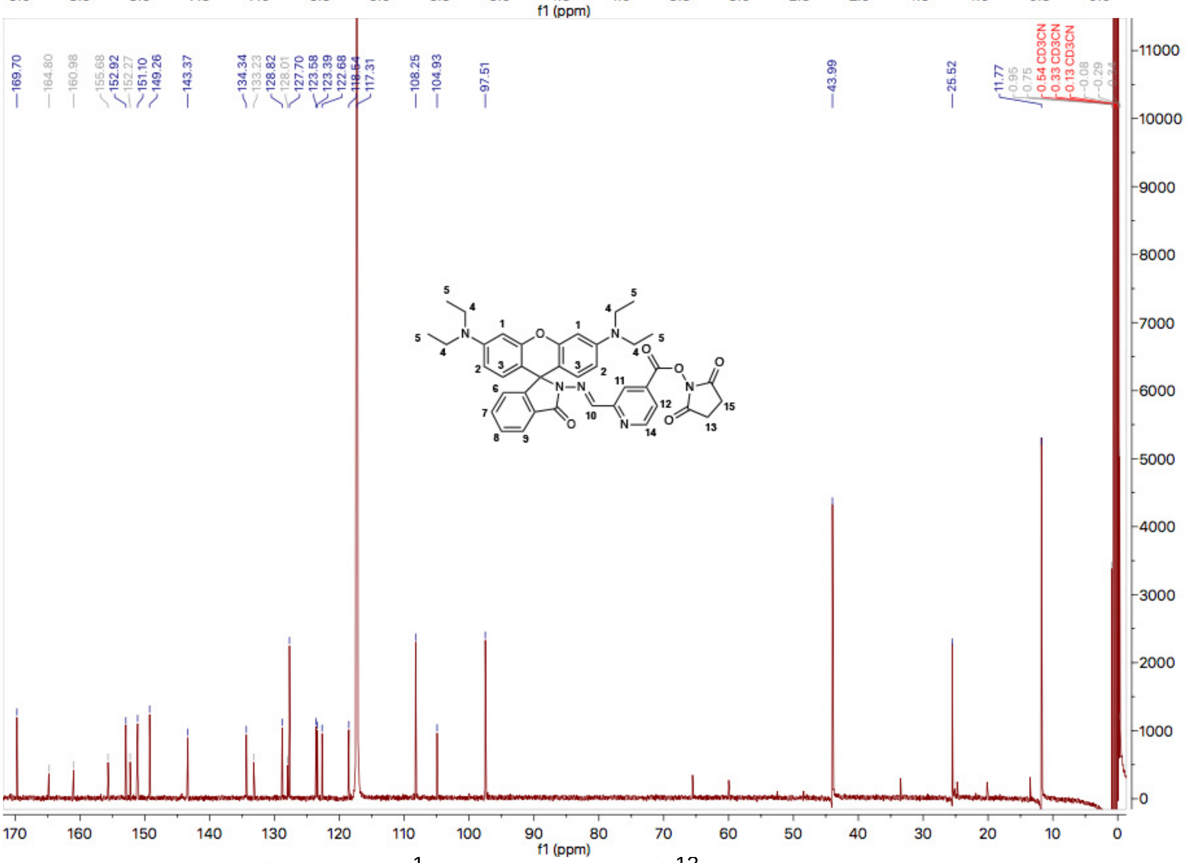

Supplementary Figure 25 | ^{1}H NMR (top) and ^{13}C NMR (bottom) spectra of **6** in CD_3CN.

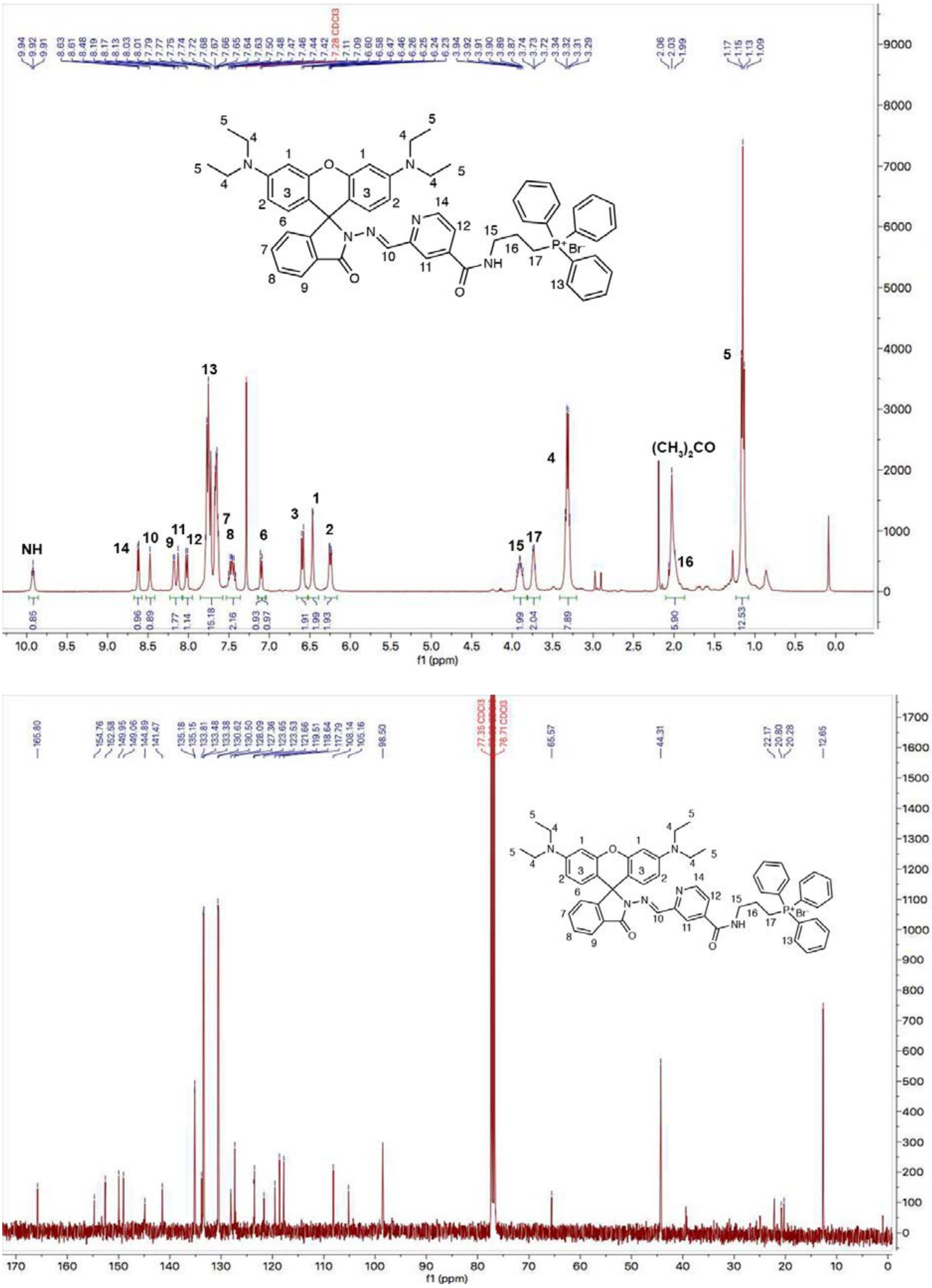

Supplementary Figure 26 | ^{1}H NMR (top) and ^{13}C NMR (bottom) spectra of MitoPFF-**1** in $CDCl_3$.

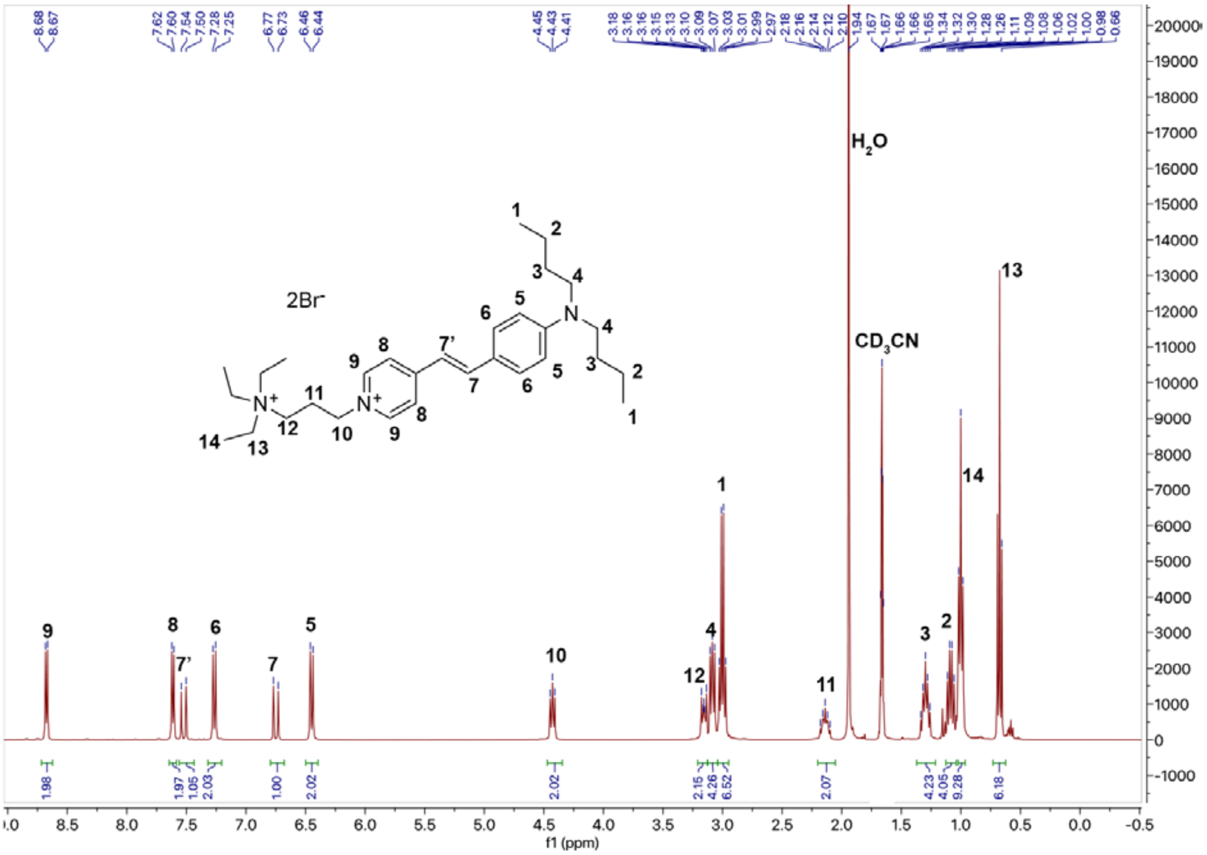

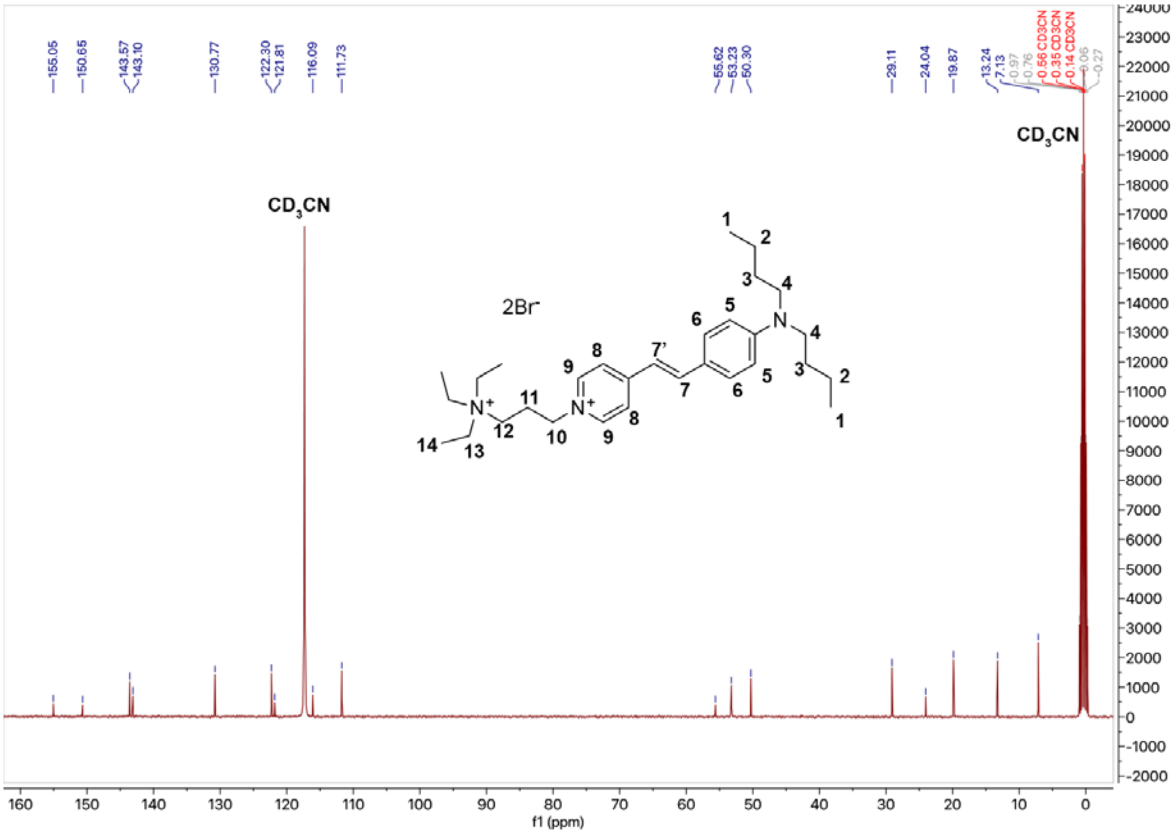

Supplementary Figure 27 | ^{1}H NMR (top) and ^{13}C NMR (bottom) spectra of FM1-43 in CD_3CN.

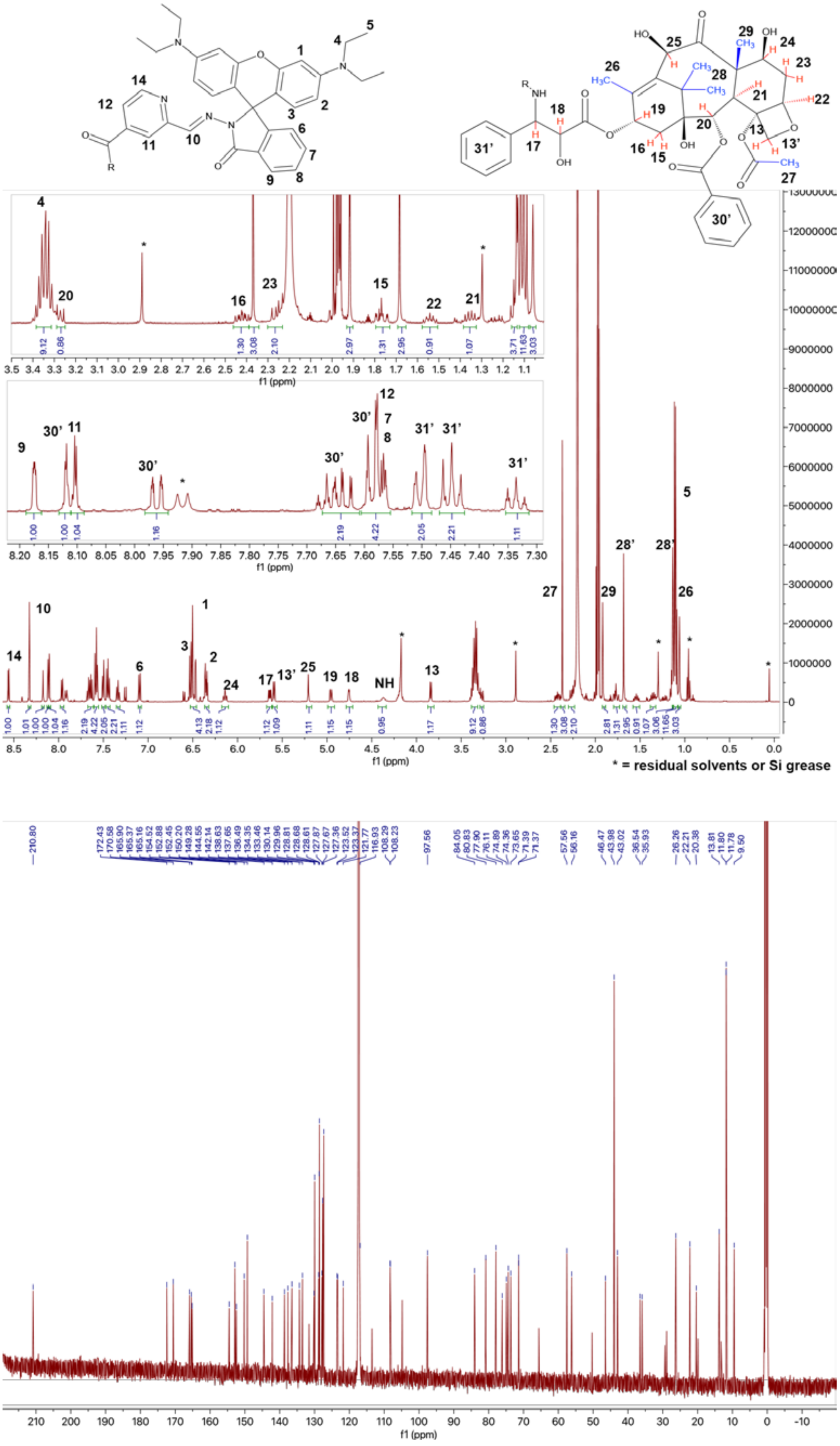

Supplementary Figure 28 | ^{1}H NMR (top) and ^{13}C NMR (bottom) spectra of TaxolPFF-**1** in CD_3CN.

Supplementary References

1. Butkevich, A. N., Lukinavicius, G., D'Este, E. & Hell, S. W. Cell-permeant large Stokes shift dyes for transfection-free multicolor nanoscopy. *J. Am. Chem. Soc.* **139,** 12378–12381 (2017).
2. Berg, S. *et al.* Design and synthesis of β-site amyloid precursor protein cleaving enzyme (BACE1) inhibitors with in vivo brain reduction of β-amyloid peptides. *J. Med. Chem.* **55,** 9346–9361 (2012).
3. Kimura, Y., Momotake, A., Takahashi, N., Kasai, H. & Arai, T. Polarity-dependent photophysical properties of hemicyanine dyes and their application in 2-photon microscopy biological imaging. *Chem. Lett.* **41,** 528–530 (2012).
4. Hassner, A., Birnbaum, D. & Loew, L. M. Charge-shift probes of membrane potential. Synthesis. *J. Org. Chem.* **49,** 2546–2551 (1984).
