# Supplementary material for: Photoregulated fluxional fluorophores for live-cell super-resolution microscopy with no apparent photobleaching
Source: Nat Commun. 2019 Mar 15;10:1232. doi: 10.1038/s41467-019-09217-7 (PMC6420572; doi:10.1038/s41467-019-09217-7)
Supplement: Supplementary file 3 — Description of Additional Supplementary Files [file 41467_2019_9217_MOESM3_ESM.pdf]

Description of Additional Supplementary Files

File Name: Supplementary Movie 1
Description: Long time-lapse (30 min) SMLM of lysosomes in a live HeLa cells applying photoactivation pulses (405 nm, 2.6 W cm–2, 20 ms) every 10 min and imaging at 561 nm (0.25 kW cm–2, 20 ms).

File Name: Supplementary Movie 2
Description: Time-lapse (13 s) SMLM of synaptic vesicles in live neurons derived from neuroblastoma cells. Imaging conditions: single photoactivation pulse (405 nm, 2.6 W cm–2, 20 ms), imaging at 561 nm (0.25 kW cm–2, 20 ms).

File Name: Supplementary Movie 3
Description: Time-lapse (19 s) SMLM of a synaptic vesicle hopping between hotspots in a projection of a live neuron. Imaging conditions: single photoactivation pulse (405 nm, 2.6 W cm–2, 20 ms), imaging at 561 nm (0.25 kW cm–2, 20 ms).

File Name: Supplementary Movie 4
Description: Time-integrated (3 s), 3D reconstruction of two hotspots (lower blobs) connected by a track through which synaptic vesicles transit. The unit cell is 1.5 µm x 1.5 µm x 1.5 µm and labeled with ticks of 0.5 µm. Imaging conditions: single photoactivation pulse (405 nm, 2.6 W cm–2, 20 ms), imaging at 561 nm (0.25 kW cm–2, 20 ms).
