# Supplementary material for: Photoregulated fluxional fluorophores for live-cell super-resolution microscopy with no apparent photobleaching
Source: Nat Commun. 2019 Mar 15;10:1232. doi: 10.1038/s41467-019-09217-7 (PMC6420572; doi:10.1038/s41467-019-09217-7)
Supplement: Supplementary file 8 — Reporting Summary [file 41467_2019_9217_MOESM8_ESM.pdf]

nature research

Corresponding author(s): Pablo Rivera-Fuentes

Reporting Summary

Nature Research wishes to improve the reproducibility of the work that we publish. This form provides structure for consistency and transparency in reporting. For further information on Nature Research policies, see Authors & Referees and the Editorial Policy Checklist.

Statistical parameters

When statistical analyses are reported, confirm that the following items are present in the relevant location (e.g. figure legend, table legend, main text, or Methods section).

n/a	Confirmed	
☐	☒	The exact sample size (n) for each experimental group/condition, given as a discrete number and unit of measurement
☐	☒	An indication of whether measurements were taken from distinct samples or whether the same sample was measured repeatedly
☐	☒	The statistical test(s) used AND whether they are one- or two-sided<br>*Only common tests should be described solely by name; describe more complex techniques in the Methods section.*
☒	☐	A description of all covariates tested
☒	☐	A description of any assumptions or corrections, such as tests of normality and adjustment for multiple comparisons
☐	☒	A full description of the statistics including central tendency (e.g. means) or other basic estimates (e.g. regression coefficient) AND variation (e.g. standard deviation) or associated estimates of uncertainty (e.g. confidence intervals)
☐	☒	For null hypothesis testing, the test statistic (e.g. F, t, r) with confidence intervals, effect sizes, degrees of freedom and P value noted<br>*Give P values as exact values whenever suitable.*
☒	☐	For Bayesian analysis, information on the choice of priors and Markov chain Monte Carlo settings
☒	☐	For hierarchical and complex designs, identification of the appropriate level for tests and full reporting of outcomes
☒	☐	Estimates of effect sizes (e.g. Cohen's d, Pearson's r), indicating how they were calculated
☐	☒	Clearly defined error bars<br>*State explicitly what error bars represent (e.g. SD, SE, CI)*

Our web collection on statistics for biologists may be useful.

Software and code

Policy information about availability of computer code

Data collection	Image acquisition: VisiVIEW (confocal) and NIS-Elements 4.30 (SMLM); Fluorescence: Origin.
Data analysis	Image analysis: ImageJ 1.51, including ThunderSTORM plugin for SMLM and MOSAIC suite for vesicle tracking; Plots, curve fitting, and statistical analysis: Prism 7; NMR: MestreNova; Cloning and sequencing visualization: SnapGene.

For manuscripts utilizing custom algorithms or software that are central to the research but not yet described in published literature, software must be made available to editors/reviewers upon request. We strongly encourage code deposition in a community repository (e.g. GitHub). See the Nature Research guidelines for submitting code & software for further information.

Data

Policy information about availability of data

All manuscripts must include a data availability statement. This statement should provide the following information, where applicable:
- Accession codes, unique identifiers, or web links for publicly available datasets
- A list of figures that have associated raw data
- A description of any restrictions on data availability

All data that support this manuscript in available upon request

Field-specific reporting

Please select the best fit for your research. If you are not sure, read the appropriate sections before making your selection.

☒ Life sciences ☐ Behavioural & social sciences ☐ Ecological, evolutionary & environmental sciences

For a reference copy of the document with all sections, see nature.com/authors/policies/ReportingSummary-flat.pdf

Life sciences study design

All studies must disclose on these points even when the disclosure is negative.

Sample size	At least three independent biological samples were measured for all experiments (different batch of cells and passage number). At least three different films, prepared in at least two different ways were used for single-molecule measurements.
Data exclusions	No data were excluded form analyses.
Replication	All experiments were repeated at least three times and all attempts at replication were successful.
Randomization	Batches of cells were assigned to experimental groups by informal randomization.
Blinding	Researchers were not blinded, but all image analyses were performed computationally and were therefore inherently unbiased.

Reporting for specific materials, systems and methods

Materials & experimental systems

n/a	Involved in the study
☐	☒ Unique biological materials
☐	☒ Antibodies
☐	☒ Eukaryotic cell lines
☒	☐ Palaeontology
☒	☐ Animals and other organisms
☒	☐ Human research participants

Methods

n/a	Involved in the study
☒	☐ ChIP-seq
☒	☐ Flow cytometry
☒	☐ MRI-based neuroimaging

Unique biological materials

Policy information about availability of materials

Obtaining unique materials	The LAMP1-mTurquoise2 plasmid has been deposited with Addgene (# 110948).

Antibodies

Antibodies used	anti-neurofilament H (NF-H) phosphorylated primary antibody (SMI31, 801602, BioLegend); mouse IgG (H+L) highly crossed-adsorbed secondary antibody (Alexa Fluor Plus 488, A32723, ThermoFisher)
Validation	Antibodies were not further validated

Eukaryotic cell lines

Policy information about cell lines

Cell line source(s)	HeLa (CCL-2) and SH-SY5Y (CRL-2266) were obtained from ATCC
Authentication	The cells lines were not further authenticated. Differentiation of SH-SY5Y was validated by immunofluorescence labeling of phosphorylated neurofilament H.
Mycoplasma contamination	HeLa cells were tested for mycoplasma and no contamination was found. SH-SY5Y were used at very early passage numbers and were not tested for mycoplasma.

Commonly misidentified lines
(See ICLAC register)

No commonly misidentified lines were used in this study.
